# Crucial roles of RSK in cell motility by catalysing serine phosphorylation of EphA2

**DOI:** 10.1038/ncomms8679

**Published:** 2015-07-09

**Authors:** Yue Zhou, Naoki Yamada, Tomohiro Tanaka, Takashi Hori, Satoru Yokoyama, Yoshihiro Hayakawa, Seiji Yano, Junya Fukuoka, Keiichi Koizumi, Ikuo Saiki, Hiroaki Sakurai

**Affiliations:** 1 Department of Cancer Cell Biology, Graduate School of Medicine and Pharmaceutical Sciences, University of Toyama, Toyama 930-0194, Japan; 2 Department of Diagnostic Pathology, Toyama University Hospital, Toyama 930-0194, Japan; 3 Division of Pathogenic Biochemistry, Institute of Natural Medicine, University of Toyama, Toyama 930-0194, Japan; 4 Division of Medical Oncology, Cancer Research Institute, Kanazawa University, Kanazawa 920-8641, Japan; 5 Department of Pathology, Nagasaki University Graduate School of Biomedical Sciences, Nagasaki 852-8501, Japan; 6 Division of Kampo Diagnostics, Institute of Natural Medicine, University of Toyama 930-0194, Toyama, Japan

## Abstract

Crosstalk between inflammatory signalling pathways and receptor tyrosine kinases has been revealed as an indicator of cancer malignant progression. In the present study, we focus on EphA2 receptor tyrosine kinase, which is overexpressed in many human cancers. It has been reported that ligand-independent phosphorylation of EphA2 at Ser-897 is induced by Akt. We show that inflammatory cytokines promote RSK-, not Akt-, dependent phosphorylation of EphA2 at Ser-897. In addition, the RSK–EphA2 signalling pathway controls cell migration and invasion of metastatic breast cancer cells. Moreover, Ser-897-phosphorylated EphA2 co-localizes with phosphorylated active form of RSK in various human tumour specimens, and this double positivity is related to poor survival in lung cancer patients, especially those with a smoking history. Taken together, these results indicate that the phosphorylation of EphA2 at Ser-897 is controlled by RSK and the RSK–EphA2 axis might contribute to cell motility and promote tumour malignant progression.

## Abstract (web summary)

The EphA2 receptor tyrosine kinase is overexpressed in many cancers and is reported to be phosphorylated by Akt. Here, Zhou *et al.* show that RSK, rather than Akt, phosphorylates EphA2 on Ser-897, and this regulates cell migration and invasion of metastatic cancer cells.

## Graphical abstract

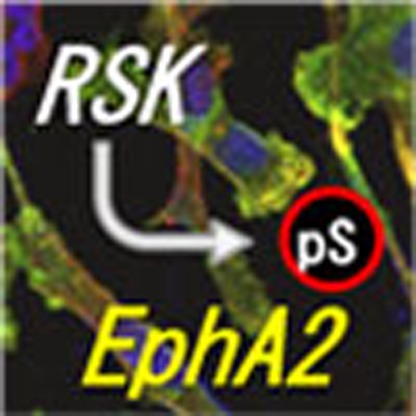

Receptor tyrosine kinases (RTKs) play central roles in human tumorigenesis and malignant progression^1^,^2^. EphA2, which belongs to the largest Eph subfamily among RTKs, regulates tissue development and maintains epithelial tissue homeostasis^3^,^4^. Overexpression of EphA2 is one of the prognostic factors in progressive tumours, including lung, breast, brain, ovarian, melanoma, prostate and urinary bladder cancers. EphA2 expression correlates with cancer metastasis, promotion of epithelial–mesenchymal transition (EMT) and maintenance of cancer stem cell properties^4^,^5^,^6^,^7^. An EphA2 tyrosine kinase inhibitor has been shown to induce tumour regression in human non-small cell lung cancer (NSCLC) xenografts *in vivo*, indicating that EphA2 is a promising molecular target in cancer therapy^8^. However, comprehensive mechanisms for receptor function remain poorly understood.

RTKs are normally activated through their own tyrosine kinase activity^9^. In addition to this classical activation model, it has become evident that the phosphorylation of serine and threonine residues plays key roles in ligand-controlled and ligand-independent functions of RTKs^10^,^11^,^12^,^13^,^14^,^15^,^16^. We have reported that tumour necrosis factor-α (TNF-α) induces the phosphorylation of epidermal growth factor receptor (EGFR) at Thr-669 in the juxtamembrane domain and Ser-1046/7 in the carboxy (C)-terminal tail through extracellular signal-regulated kinase (ERK) and p38 pathways, respectively^11^,^12^,^13^. Ser-1046/7 is involved in clathrin-mediated endocytosis of EGFR and Thr-669 phosphorylation causes negative feedback regulation of its tyrosine kinase. Similarly, three serine residues in the C-tail of fibroblast growth factor receptor 1 are phosphorylated by ERK, protein kinase Cɛ (PKCɛ) and p90 ribosomal S6 kinase 2 (RSK2), which are critical for negative feedback regulation and endocytosis of the receptor^14^,^15^,^16^. Hence, study on serine/threonine phosphorylation is essential to understand fully the roles of RTKs in pathogenic alterations of cancers, but we are still far from clarifying the whole picture.

Recently, increasing evidence has shown that upregulation of EphA2 along with downregulation of its ligands, including ephrin-A1, disrupts orderly epithelial adhesion of cancer cells, suggesting ligand-independent functions of EphA2 in tumour microenvironments^4^,^17^. Indeed, Miao *et al.*^18^ demonstrated an alternative function of EphA2, in which ligand-independent Akt phosphorylation of EphA2 at Ser-897 promotes growth factor-induced cell polarization, lamellipodium protrusion and cell migration, and is correlated with the tumour grade of human astrocytoma. In contrast, ligand-dependent EphA2 tyrosine kinase activation inhibited cell migration and invasion. A large number of serine and threonine residues capable of being phosphorylated have been identified by mass spectral analysis^19^; therefore, further characterization of EphA2 phosphorylation is essential for understanding the ligand-independent functions of EphA2 in cancer cells.

In the present study, we try to determine whether an inflammatory cytokine promotes EphA2 phosphorylation at Ser-897. We show that TNF-α induces Ser-897 phosphorylation, but, unexpectedly, it is directly regulated by the ERK–RSK signalling pathway, but not by the PI3K–Akt pathway. Therefore, we further characterize activation of the RSK–EphA2 pathway by other factors activating ERK and their roles in regulating the cell motility of human cancer cells. Furthermore, we investigate the relationship between this pathway and clinical outcomes using human lung cancer tissue specimens.

## Results

### Phosphorylation of EphA2 at Ser-897 is induced by TNF-α

**Figure 1:**
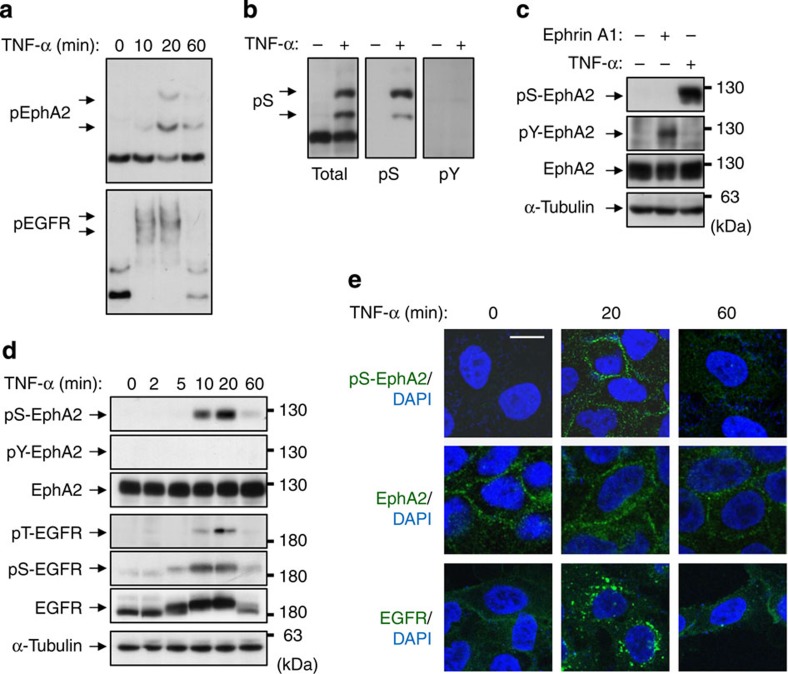
Phosphorylation of EphA2 at Ser-897 is induced by TNF-α stimulation. (**a**) Whole-cell lysates from HeLa cells treated with TNF-α (20 ng ml^−1^) for 10, 20 and 60 min were separated by Zn^2+^-Phos-tag SDS–PAGE and immunoblotted with anti-EphA2 and EGFR antibodies. (**b**) Whole-cell lysates from HeLa cells treated with TNF-α for 20 min were separated by Zn^2+^-Phos-tag SDS–PAGE and immunoblotted with anti-EphA2, pS-EphA2 and pY-EphA2. (**c**) Whole-cell lysates from HeLa cells treated with ephrin-A1 (100 ng ml^−1^) for 10 min or TNF-α for 20 min were separated by normal SDS–PAGE and immunoblotted with anti-pS-EphA2, pY-EphA2, EphA2 and α-tubulin antibodies. (**d**) HeLa cells were stimulated with TNF-α for the indicated periods. Whole-cell lysates were electrophoresed and probed with primary antibodies against pS-EphA2, pY-EphA2, EphA2, pT-EGFR, pS-EGFR, EGFR and α-tubulin. (**e**) HeLa cells were stimulated with TNF-α for 20 and 60 min. After fixation and permeabilization, cells were immunofluorescently stained with pS-EphA2, EphA2 or EGFR (clone LA1). Scale bar, 20 μm. Shown are representative images from three independent experiments.

In our previous studies, we detected TNF-α-induced Ser/Thr phosphorylation of EGFR^12^. To determine whether TNF-α also induces the phosphorylation of EphA2, Zn^2+^-Phos-tag SDS–polyacrylamide gel electrophoresis (SDS–PAGE)^20^ that is an approach for the detection and separation of phosphorylated proteins, was used (Fig. 1a). First, we analysed the phosphorylation of EphA2 and EGFR in HeLa cells using Phos-tag SDS–PAGE. Several shifted bands of EGFR were clearly detected upon TNF-α stimulation, indicating different phosphorylation states. Similarly, but slightly delayed, two major shifted bands of EphA2 were observed. These results indicate that, in parallel with EGFR phosphorylation, EphA2 is targeted by an inflammatory signalling pathway. We next used phospho-Ser-897 EphA2 (pS-EphA2) antibody and phospho-Tyr-588 EphA2 (pY-EphA2) antibody in Phos-tag SDS–PAGE (Fig. 1b). Upon stimulation with TNF-α, pS-EphA2 antibody recognized two major bands that showed similar gel retardation in the total EphA2 blot, whereas no band with a similar shift appeared in the pY-EphA2 blot.

In normal SDS–PAGE, TNF-α and ephrin-A1 selectively induced pS-EphA2 and pY-EphA2, respectively (Fig. 1c). Selectivity of pS-EphA2 antibody to the phosphorylation of Ser-897 was confirmed in HEK293 cells expressing Ser-897 to Ala substitution mutant EphA2 (SA) (Supplementary Fig. 1a). Moreover, pY-EphA2 was not induced until 60 min after TNF-α stimulation, although pS-EphA2 was promoted from 10 min and largely reduced at 60 min (Fig. 1d). This time course was similar to that of the phosphorylation of EGFR at Thr-669 and Ser-1046/7 (Fig. 1d). In addition, TNF-α and interleukin-1β (IL-1β) induced pS-EphA2 in human lung adenocarcinoma A549 cells (Supplementary Fig. 1b). We previously demonstrated that TNF-α promotes EGFR endocytosis in a p38-dependent manner^11^,^12^. Immunofluorescence analysis demonstrated that EphA2 was not internalized and pS-EphA2 was localized on the cell surface (Fig. 1e). Altogether, our results clearly demonstrated that TNF-α induces the phosphorylation of EphA2 at Ser-897 in a tyrosine kinase activity-independent manner.

### Akt does not control Ser-897 phosphorylation of EphA2

**Figure 2:**
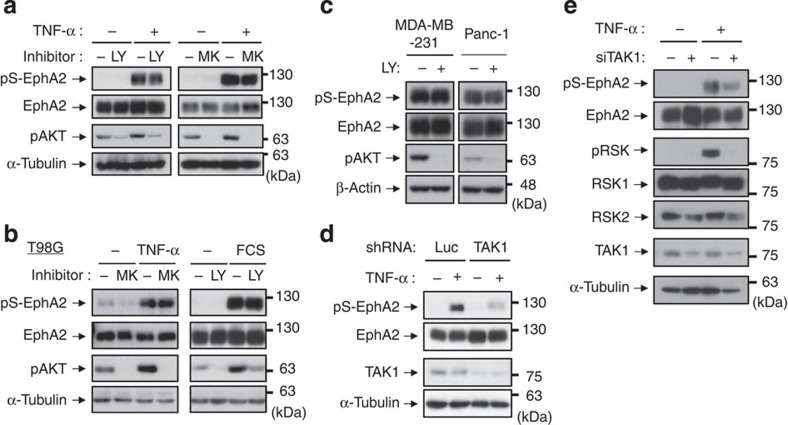
The phosphorylation of EphA2 at Ser-897 is induced by TAK1, but not by Akt. (**a**,**b**) HeLa (**a**) or T98G (**b** left) cells were pre-treated with LY294002 (10 μM) or MK-2206 (10 μM) for 30 min and then stimulated with TNF-α for 20 min. T98G cells were starved using FCS-free medium for 24 h, treated with LY294002 for 30 min and then treated with 10% FCS for 10 min (**b**, right). (**c**) MDA-MB-231 and Panc-1 cells were treated with LY294002 for 30 min. (**d**) HeLa cells stably transfected shRNA expression vectors against luciferase and TAK1 were stimulated with TNF-α for 20 min. (**e**) HeLa cells were transfected with siRNAs against TAK1 or negative control. At 72 h post transfection, cells were treated with TNF-α for 20 min. Whole-cell lysates were immunoblotted with anti-pS-EphA2, EphA2, pAKT, pRSK, RSK1, RSK2, TAK1, β-actin and α-tubulin antibodies.

Miao *et al.*^18^ previously reported that Akt directly induces EphA2 phosphorylation at Ser-897 in glioma cells. Consequently, we investigated whether Akt is also involved in pS-EphA2 in HeLa cells. Cells were pre-incubated with PI3K inhibitor (LY294002) or an allosteric Akt inhibitor (MK-2206) for 30 min and then stimulated with TNF-α for 20 min (Fig. 2a). Unexpectedly, pS-EphA2 was not inhibited by these inhibitors, while Akt phosphorylation was significantly inhibited. We also confirmed these results using immunofluorescence analysis (Supplementary Fig. 2a). As used by Miao *et al.*, we next attempted to evaluate pS-EphA2 in T98G human glioblastoma cells stimulated with TNF-α or FCS (Supplementary Fig. 2b). As shown in Fig. 2b, MK-2206 and LY294002 abolished pS-EphA2 in neither TNF-α stimulation nor FCS stimulation. Moreover, constitutively present pS-EphA2 in MDA-MB-231 human breast cancer cells harbouring *KRAS* and *BRAF* mutations and Panc-1 human pancreatic cancer cells carrying *KRAS* mutation was also resistant to PI3K inhibition (Fig. 2c). Collectively, these results demonstrate that the phosphorylation of EphA2 at Ser-897 is not catalysed by Akt.

### TAK1 controls TNF-α-induced phosphorylation of EphA2

The results for the PI3K–Akt pathway as shown above are reasonable because we detected only slight activation of Akt in TNF-α-treated HeLa cells (Fig. 2a). By contrast, transforming growth factor-β-activated kinase 1 (TAK1) is a key kinase in the TNF-α and IL-1β signalling pathway leading to MAPK and NF-κB activation^21^. RNAi knockdown experiments using shRNA or siRNA against TAK1 demonstrated that TAK1 is essential for TNF-α-induced pS-EphA2 (Fig. 2d,e). In addition, overexpression of EphA2 with activated TAK1 in HeLa cells caused an increase in EphA2 phosphorylation (Supplementary Fig. 2c). These results indicate that EphA2 is phosphorylated by downstream kinases of TAK1.

### RSK inhibitor blocks phosphorylation of EphA2 at Ser-897

**Figure 3:**
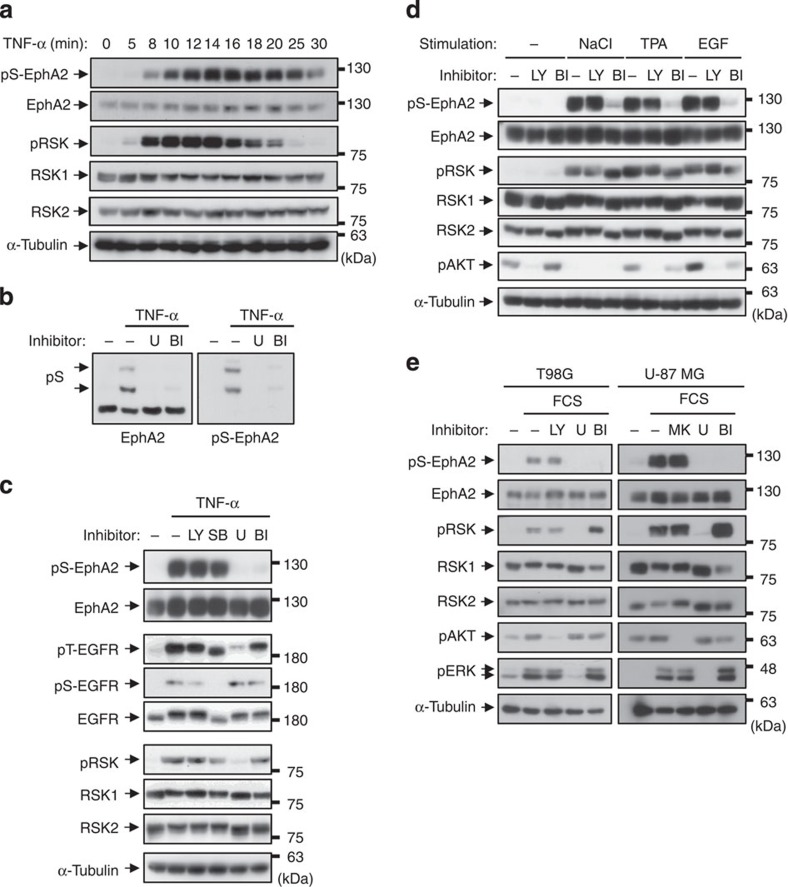
Phosphorylation of pS-EphA2 is induced by RSK. (**a**) HeLa cells were stimulated with TNF-α for the indicated periods. Whole-cell lysates were immunoblotted with anti-pS-EphA2, EphA2, pRSK, RSK1, RSK2 and α-tubulin antibodies. (**b**,**c**) Whole-cell lysates from HeLa cells pre-treated with LY294002 (10 μM), SB203580 (10 μM), U0126 (5 μM) or BI-D1870 (10 μM) for 30 min and then stimulated with TNF-α for 20 min were separated by Zn^2+^-Phos-tag SDS–PAGE and immunoblotted with anti-EphA2 and pS-EphA2 antibodies (**b**), or by normal SDS–PAGE and immunoblotted with anti-pS-EphA2, EphA2, pT-EGFR, pS-EGFR, EGFR, pRSK, RSK1, RSK2 and α-tubulin antibodies (**c**). (**d**) HeLa cells were pre-treated with LY294002 or BI-D1870 for 30 min and then stimulated with NaCl (0.3 M), TPA (100 ng ml^−1^) or EGF (10 ng ml^−1^) for 10 min. Whole-cell lysates were immunoblotted with primary antibodies against pS-EphA2, EphA2, pRSK, RSK1, RSK2, pAKT and α-tubulin. (**e**) T98G and U-87 MG cells starved in FCS-free medium for 24 h were treated with LY294002, MK-2206, U0126 and BI-D1870 for 30 min and then stimulated with 10% FCS for 10 min. Whole-cell lysates were immunoblotted with primary antibodies against pS-EphA2, EphA2, pRSK, RSK1, RSK2, pAKT, pERK and α-tubulin.

To identify the kinases responsible for pS-EphA2, we obtained the substrate sequence LOGO of Ser/Thr kinases from the PhosphoSitePlus database ( http://www.phosphosite.org/homeAction.do)^19^. Among Ser/Thr kinases, the LOGOs of RSK1 and RSK2, downstream kinases of ERK, are similar to that of Akt. Akt and RSKs are members of the AGC family kinases that share substrate specificity characterized by Arg at position -3 relative to the phosphorylated Ser/Thr^19^,^22^,^23^; therefore, we next qualified RSK as a putative candidate for the kinase responsible for Ser-897 phosphorylation. As shown in Fig. 3a, TNF-α-induced pS-EphA2 was induced from 8 min, peaked at 14 min and was then gradually downregulated, which closely correlated with the time course of pRSK. Pretreatment with MEK inhibitor (U0126) or RSK inhibitor (BI-D1870) abrogated the appearance of shifted bands in Phos-tag SDS–PAGE and pS-EphA2 in normal SDS–PAGE as well as pS-EphA2 staining in immunofluorescence, suggesting that the ERK–RSK pathway controls pS-EphA2 (Fig. 3b,c, and Supplementary Fig. 3a). We previously demonstrated that Thr-669 phosphorylation of EGFR is also induced by the ERK pathway^12^,^13^; however, it was inhibited by U0126 but not by BI-D1870 (Fig. 3c), indicating that different kinases in the ERK pathway control pS-EphA2 and pT-EGFR. Moreover, we tried to examine the effects of various other stimuli that activate RSK, including high osmotic stress (0.3 M NaCl), 12-*o*-tetradecanoylphorbol 13-acetate (TPA) and EGF. As expected, pS-EphA2 was strongly induced by all of these agents and it was inhibited by BI-D1870, but not by LY294002 (Fig. 3d). U0126 and BI-D1870 also blocked EphA2 phosphorylation in TNF-α-stimulated A549 cells (Supplementary Fig. 3b), in FCS- or TNF-α-stimulated T98G and U-87 MG cells (Fig. 3e and Supplementary Fig. 3c) and in MDA-MB-231 and Panc-1 cells (Supplementary Fig. 3d). BI-D1870 enhanced phosphorylation of RSK in some cell lines, including T98G and MDA-MB-231 cells (Fig. 3e and Supplementary Fig. 3d), possibly due to inhibition of the downstream negative feedback regulation^24^. However, phosphorylation of Bad at Ser-112, an RSK substrate^25^, was inhibited, indicating that BI-D1870 substantially inhibited RSK kinase activity (Supplementary Fig. 3e). Collectively, these results demonstrate that the phosphorylation of EphA2 at Ser-897 is induced by RSK or its downstream kinases.

### EphA2 at Ser-897 is directly phosphorylated by RSK

**Figure 4:**
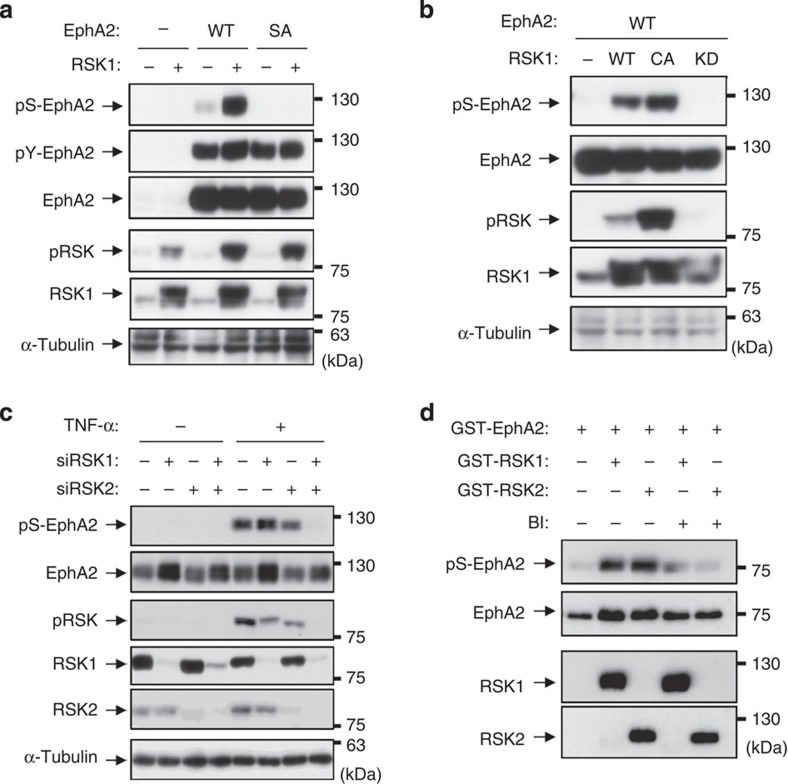
EphA2 at Ser-897 is phosphorylated by RSK1/2. (**a**,**b**) HEK293 cells were transfected with expression vectors for EphA2, RSK1 and its substitution mutants. At 24 h post transfection, whole-cell lysates were immunoblotted with anti-pS-EphA2, pY-EphA2, EphA2, pRSK, RSK1 and α-tubulin antibodies. (**c**) HeLa cells were transfected with siRNAs against RSK1, RSK2 or negative control. At 72 h post transfection, cells were stimulated with TNF-α for 20 min. Whole-cell lysates were immunoblotted with primary antibodies against pS-EphA2, EphA2, pRSK, RSK1, RSK2 and α-tubulin. (**d**) Recombinant human GST-EphA2 was incubated with recombinant human active GST-RSK1 or RSK2 in the absence or presence of BI-D1870 (0.1 μM) at 30 °C for 30 min. The reaction mixtures were analysed by immunoblotting with anti-pS-EphA2, EphA2, RSK1 and RSK2 antibodies.

To identify RSK as the kinase responsible for pS-EphA2, HEK293 cells were transfected with EphA2 and RSK1. Coexpression of EphA2 with RSK1 induced Ser-897 phosphorylation of wild-type EphA2 but not its SA mutant (Fig. 4a). Activation of TAK1 caused RSK phosphorylation as well as pS-EphA2 (Supplementary Fig. 2c). In addition, constitutively active RSK1 but not kinase-dead RSK1 induced pS-EphA2, indicating that the kinase activity of RSK1 is indispensable for pS-EphA2 (Fig. 4b).

Among the four members of the RSK family, RSK1 and RSK2 are major isoforms involved in cancer metastasis and EMT^26^,^27^; therefore, we carried out an RNAi experiment on RSK1 and RSK2. As shown in Fig. 4c, single knockdown of neither RSK1 nor RSK2 reduced pS-EphA2, but RSK1/2 double knockdown attenuated pS-EphA2 completely. Moreover, pS-EphA2 was partially recovered by RSK1 re-expression (Supplementary Fig. 4), suggesting that pS-EphA2 is regulated redundantly by RSK1 and RSK2. Consequently, to obtain direct evidence for the catalysis of EphA2 phosphorylation, we performed an *in vitro* kinase assay using recombinant kinases and found that both GST-RSK1 and GST-RSK2 phosphorylated Ser-897 of GST-EphA2 (Fig. 4d). Collectively, these results demonstrate that the phosphorylation of Ser-897 is catalysed by RSK1/2 directly.

### RSK–EphA2 axis is involved in cell motility

**Figure 5:**
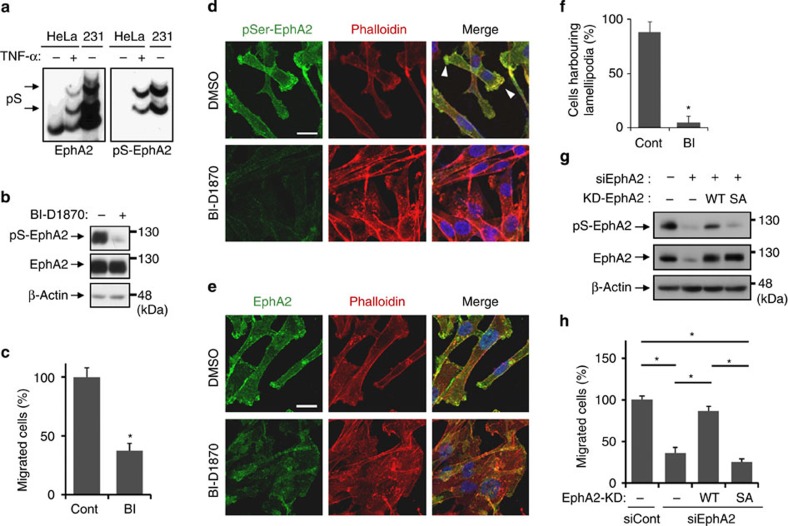
The RSK–EphA2 axis controls cell motility. (**a**) Whole-cell lysates from HeLa cells treated with TNF-α for 20 min or untreated MDA-MB-231 cells were separated by Zn^2+^-Phos-tag SDS–PAGE and immunoblotted with anti-EphA2 antibody. (**b**–**f**) MDA-MB-231 cells were pre-treated with BI-D1870 (10 μM) for 30 min and then scratched with a pipette tip. After 48 h of incubation, whole-cell lysates were immunoblotted with primary antibodies against pS-EphA2, EphA2 and β-actin (**b**) Migrated cells were counted manually under a microscope (**c**) Data are the means±s.d. of at least three fields. Similar results were obtained in at least three independent experiments. *P<0.05 by Student's *t*-test. At the same time, the migration border cells were immunofluorescently stained with anti-pS-EphA2 or EphA2 antibodies (**d**,**e**) and cells harbouring lamellipodia were counted manually under a microscope (**f**) Scale bar, 20 μm. Data are the means±s.d. of at least three fields. Similar results were obtained in at least three independent experiments. *P<0.05 by Student's *t*-test. (**g**,**h**) MDA-MB-231 cells were transfected with siRNA against EphA2 or negative control and EphA2 mutation-expression plasmids. The immunoblotting results from whole-cell lysates with anti-pS-EphA2, EphA2 and β-actin antibodies are shown in **g** and the results of scratch assay are shown in **h**. Data are the means±s.d. of at least three fields. Similar results were obtained in at least three independent experiments. *P<0.05 by analysis of variance followed by Tukey–Kramer HSD test.

It has been reported that Ser-897 phosphorylation of EphA2 promotes cell migration and invasion^18^. RSK1 and RSK2 are also known as key kinases for metastatic properties in various types of cancer cell^26^,^27^; therefore, we tried to determine whether the novel RSK–EphA2 axis induces cell motility. MDA-MB-231 cells, in which the RSK–EphA2 axis is constitutively activated (Fig. 5a), were adopted for a scratch assay. Treatment of RSK inhibitor BI-D1870 continuously inhibited pS-EphA2 for 48 h (Fig. 5b). We confirmed that there were no significant differences in cell proliferation and cell death between BI-D1870-treated cells and control cells (Supplementary Fig. 5a and b). Although cells migrated to the scratched area in the control sample, significant attenuation of cell migration was observed in BI-D1870-treated cells (Fig. 5c). Similar results were obtained using siRNAs against RSK1 and RSK2 (Supplementary Fig. 5c and d). Immunofluorescence staining on the migration border demonstrated that pS-EphA2 and EphA2 were preferentially localized in the migrating front with F-actin in lamellipodia in control cells (Fig. 5d,e). On the other hand, BI-D1870 not only inhibited staining of pS-EphA2 but also collapsed the elongated and polarized morphology. In addition, the formation of lamellipodia was notably interfered with and EphA2 diffused all over the cells by the inhibition of RSK activity (Fig. 5e,f). Moreover, BI-D1870 and RSK1/2 knockdown reduced invasive ability in Matrigel-coated chamber (Supplementary Fig. 5e and f). These results suggested that RSK1/2 control cell motility by maintaining pS-EphA2 localization at the edge in the direction of movement, such as in lamellipodia.

To provide direct evidence for the role of the RSK–EphA2 axis in cell motility, the expression of EphA2 in EphA2-knockdown cells was restored by the transfection of siRNA-resistant complementary DNA encoding EphA2 (Fig. 5g,h). Cell migration reduced by EphA2 knockdown was recovered by re-expression of kinase-dead EphA2, but not that of its S897A mutant. In addition, TNF-α-induced migration of A549 cells was inhibited by BI-D1870 or EphA2 knockdown (Supplementary Fig. 6a and b). Collectively, these results indicate that RSK-mediated Ser-897 phosphorylation of EphA2 is indispensable for cell motility in a tyrosine kinase activity-independent fashion.

### Molecular-targeted agents inhibited EphA2 phosphorylation

**Figure 6:**
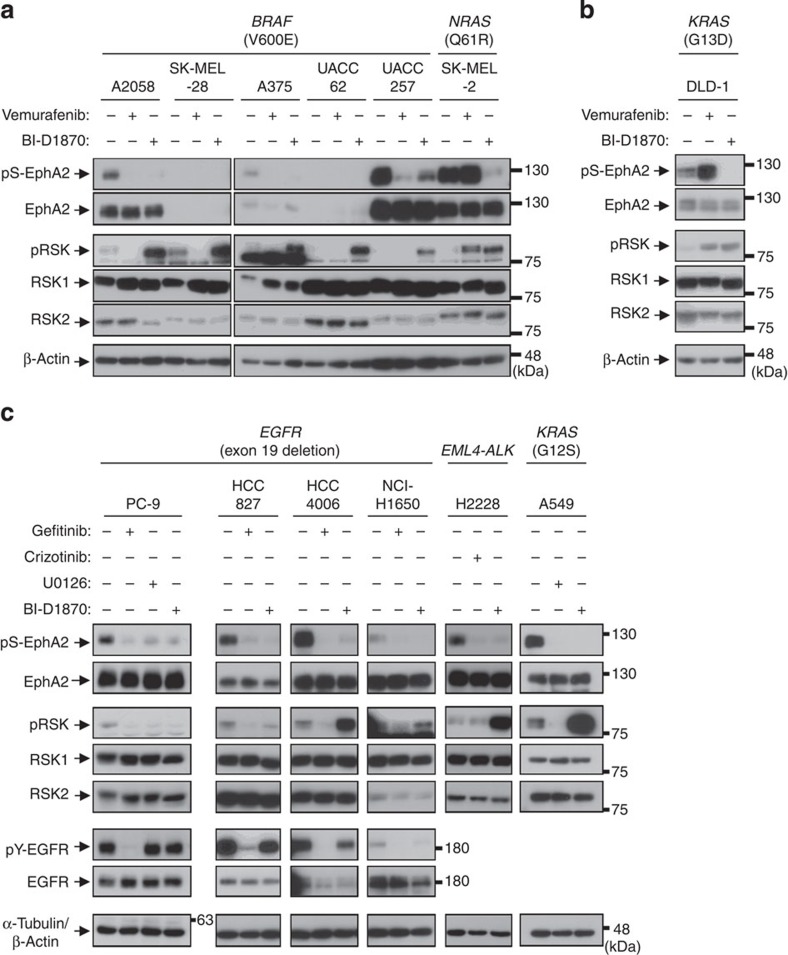
Molecular-targeted agents inhibited pS-EphA2. (**a**) Human melanoma cells (A2058, SK-MEL-28, A375, UACC62, UACC257 and SK-MEL-2), (**b**) DLD-1 colon cancer cells and (**c**) lung adenocarcinoma cells (PC-9, HCC827, HCC4006, NCI-H1650, H2228 and A549) were treated with vemurafenib (1 μM), BI-D1870 (10 μM), gefitinib (1 μM), crizotinib (10 μM) or U0126 (5 μM) for 30–60 min. Whole-cell lysates were immunoblotted with primary antibodies against pS-EphA2, EphA2, pRSK, RSK1, RSK2, pY-EGFR, EGFR, β-actin and α-tubulin.

A large proportion of driver oncogene products are known to activate the ERK signalling pathway constitutively to induce cell proliferation and metastatic properties^28^,^29^. First, we studied human melanoma cell lines with *BRAF*-V600E mutation (A2058, SK-MEL-28, A375, UACC62 and UACC257) and *NRAS*-Q61R mutation (SK-MEL-2). Among these melanoma cell lines, A2058, A375, UACC257 and SK-MEL-2 expressed total EphA2 protein and pS-EphA2 constitutively (Fig. 6a). Vemurafenib, a BRAF inhibitor, reduced pS-EphA2 in cells harbouring *BRAF* mutation, but rather increased pS-EphA2 in *NRAS*-mutated SK-MEL-2 cells (Fig. 6a) as well as in *KRAS*-mutated DLD-1 human colorectal cancer cells (Fig. 6b). Paradoxical upregulation of pS-EphA2 in *RAS*-mutated cells might be controlled by the activation of CRAF^30^,^31^. Trametinib, an MEK inhibitor used for the treatment of melanoma in the clinical setting^32^, also inhibited pS-EphA2 (Supplementary Fig. 7). Most importantly, RSK inhibitor BI-D1870 abrogated pS-EphA2 in all these cell lines (Fig. 6a,b).

We next analysed pS-EphA2 in human NSCLCs with *EGFR* exon 19 deletion (PC-9, HCC827, HCC4006 and NCI-H1650), *EML4-ALK* fusion (H2228) and *KRAS* mutation (A549). BI-D1870, U0126 and each targeted tyrosine kinase inhibitor, including gefitinib and crizotinib, abrogated pS-EphA2, indicating that the RSK–EphA2 axis was constitutively activated by driver oncogene products in all these lung cancer cells (Fig. 6c). Overall, these results highlight that the RSK–EphA2 axis is under the control of driver oncogenes, and suggest that molecular-targeted agents have the potential to block cancer migration and invasion via inhibition of the RSK–EphA2 pathway.

### Immunohistochemical co-localization of pS-EphA2 and pRSK

**Figure 7:**
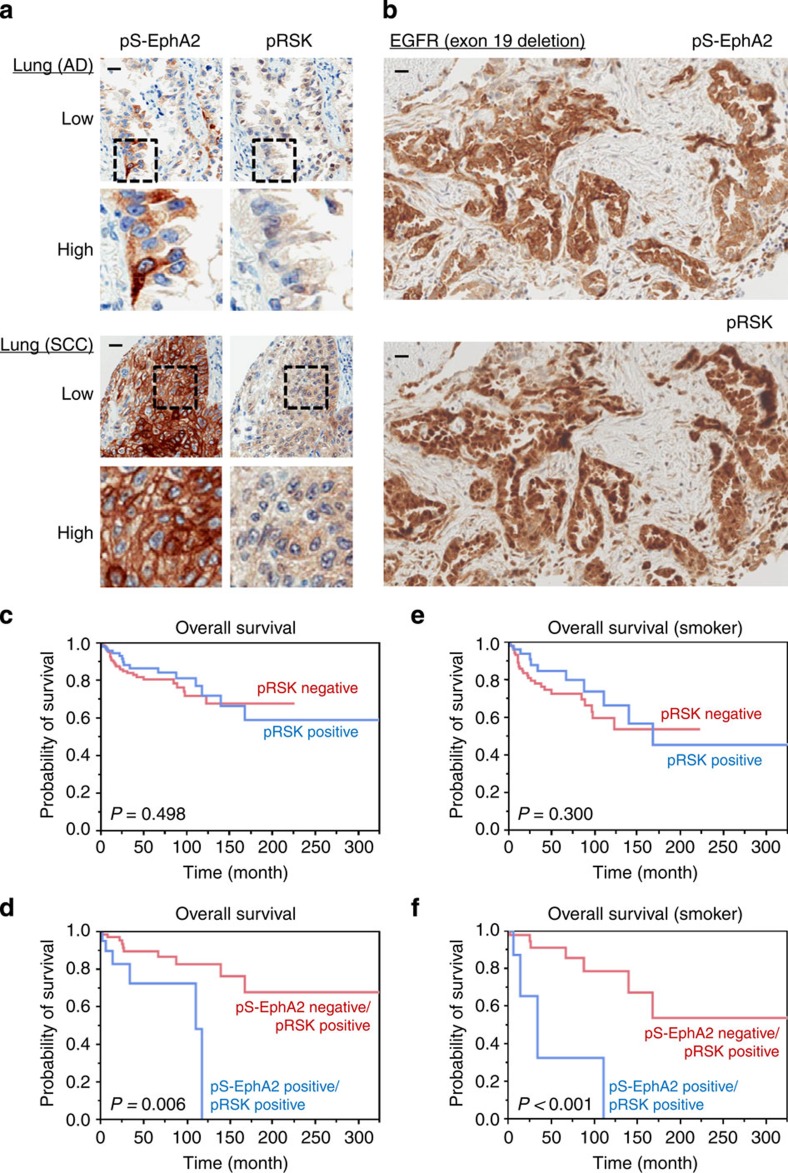
pS-EphA2 and pRSK are co-localized in cancer patients' specimens and the RSK–EphA2 axis is associated with the overall survival of lung cancer patients. (**a**) A multi-cancer tissue microarray, including 1,010 cores from 13 organ cancer tissues, was adopted for immunohistochemical staining using primary antibodies against pS-EphA2 and pRSK. Typical staining images of lung cancer tissues, including adenocarcinoma (AD) and squamous cell carcinoma (SCC), at low- and high-power magnifications are shown. Scale bar, 20 μm. (**b**) Typical immunohistochemical staining of pS-EphA2 and pRSK in *EGFR*-mutated (exon 19 deletion) lung adenocarcinoma tissues are shown. Scale bar, 20 μm. (**c**–**f**) Postoperative overall Kaplan–Meier survival curves of all the lung cancer patients (**c**,**d**) or smoking patients (**e**,**f**) were compared according to pRSK negativity or positivity (**c**,**e**) or pS-EphA2/pRSK double positivity (**d**,**f**) *P* values were calculated by the log-rank tests.

We immunohistochemically investigated pRSK and pS-EphA2 using a multi-cancer tissue microarray, which included 1,010 cores from 13 organ cancer tissues. We found double-positive samples in various cancers except for one stomach sample exhibiting pS-EphA2 positivity and pRSK negativity, suggesting a strong signalling correlation between EphA2 and RSK in human cancer tissues (Supplementary Fig. 8a–f). Figure 7a showing typical specimens of lung adenocarcinoma and squamous cell carcinoma indicates that pS-EphA2 and pRSK were potentially co-localized. Almost completely matched expression of pS-EphA2 and pRSK was also observed in lung adenocarcinoma tissues with activating EGFR mutations, including exon 19 deletion (Fig. 7b and Supplementary Fig. 8g). High-power magnifying images demonstrated that pS-EphA2 was stained mainly in the cell membrane, but also in the cytoplasm (Fig. 7a and Supplementary Fig. 8g). On the other hand, pRSK was located in both cytoplasm and nucleus (Fig. 7a and Supplementary Fig. 8g). Moreover, selective luminal membrane-side staining was an interesting observation in colon cancer tissues (Supplementary Fig. 8a). Collectively, these results suggest functional correlation between the serine phosphorylation of EphA2 and the activation of RSK in human tumour microenvironments.

### Poor prognosis of pS-EphA2/pRSK double-positive patients

It has been demonstrated that EphA2 protein expression is increased in smokers and predicts poor survival in NSCLCs^33^. Therefore, we finally explored the role of the RSK–EphA2 pathway in patients' prognosis using a lung cancer tissue microarray, which consists of a total of 353 samples, including 175 adenocarcinomas, 88 squamous cell carcinomas and others. There were no significant differences between clinicopathological factors, including smoking, and the expression of pS-EphA2/pRSK (Supplementary Table). Although there was no significant difference between pRSK expression (Fig. 7c,e) or total RSK1 expression (Supplementary Fig. 9a and b) and overall survival both in all patients and in patients with a smoking history, pS-EphA2/pRSK double-positive patients had poorer survival duration than the pS-EphA2-negative/pRSK-positive group (Fig. 7d). Moreover, a drastic difference was observed in smoking patients (Fig. 7f). These results indicate that RSK-mediated serine phosphorylation of EphA2 is involved in poorer overall survival in lung cancer.

## Discussion

The phosphorylation of EphA2 at Ser-897 has been characterized as an important reaction regulating tumour progression of human glioma^18^. Akt has been identified to be responsible for ligand-independent migration and invasion by inducing Ser-897 phosphorylation, in which phospho-(Ser/Thr) Akt substrate antibody that recognizes [RXXpS/pT] has mainly been used to detect the phosphorylated protein. EphA2 amino acids 894–897 [RLPpS] completely match the consensus sequence; however, it is also a putative substrate of RSK, as shown in the PhosphoSitePlus database^19^. Indeed, we report here that RSK1 and RSK2, but not Akt, catalyse Ser-897 phosphorylation directly in cytokine, growth factor and oncogenic signalling pathways in all of the human cancer cells that we tested, including glioma cells. In addition, the immunohistochemical co-localization of pS-EphA2 and activated RSK supports the existence of a novel RSK–EphA2 signalling pathway in tumour microenvironments. Nonetheless, it is still essential to consider carefully the contribution of Akt in EphA2 phosphorylation because Akt is reported to activate ERK in some conditions such as stimulation with platelet-derived growth factor.

Two major pSer-897 bands were detected in immunoblot results using Phos-tag SDS–PAGE (Fig. 1a,b, Figs 3b and 5a). These results demonstrate that there is at least one additional phosphorylation site other than Ser-897 in EphA2. In the PhosphoSitePlus database, more than 10 serine and threonine residues are deposited as putative phosphorylation sites in the intracellular domain of human EphA2 (ref. 19). In particular, the amino-acid sequence around Ser-897 (SIRLPS^897^TSGS) contains several serine and threonine residues. Therefore, we are now trying to identify new phosphorylation sites and their responsible kinases to understand fully the tyrosine kinase- and ligand-independent prometastatic function of EphA2. In addition, the amino acid corresponding to Ser-897 and surrounding amino acids are largely conserved in EphA1, another RTK of the Eph receptor family, which is also involved in invasion, metastasis and poor prognosis of cancer patients^34^,^35^. Therefore, although the level of EphA1 expression in human cancer cells used in this study is very low (Supplementary Fig. 10), it is essential to characterize whether the serine residue of EphA1 is a substrate for RSK or Akt.

RSK has been reported to induce cell proliferation through promoting cell cycle progression and cell survival through modulating activity of the BCL2 family in various malignancies^26^. The ERK–RSK pathway has also been extensively studied in terms of effects that promote cancer migration, invasion, metastasis and EMT both *in vitro* and *in vivo*^27^. For example, Doehn *et al.*^36^ reported that treatment with several RSK inhibitors, including BI-D1870, reduced migration as well as blocked TGF-β/TNF-α-induced, ERK-dependent EMT through inhibition of the FRA1- and c-Jun-dependent transcriptional programme of prometastatic genes, including those encoding extracellular cell matrix (ECM) components and matrix metalloproteinases (MMPs). In the present study, RSK1/2 were shown to have redundant roles in promoting both EphA2 phosphorylation and cell migration, supporting the metastasis-promoting ability of RSK1/2.

In addition, RSK can regulate cell migration by suppressing integrin-mediated cell adhesion through phosphorylation of the scaffolding protein filamin A at Ser-2125 (refs 37, 38). Interestingly, EphA2 on the cell surface can also interact with integrins to regulate cell adhesion. Moreover, both RSK and EphA2 have been demonstrated to control RhoA and RhoG small GTPase activities^27^,^39^,^40^,^41^. In particular, Kawai *et al.*^42^ reported that pS-EphA2 promotes the interaction of EphA2 with ephexin4 to induce the activation of RhoG. Consequently, our new finding on the functional connection between RSK and EphA2 encourages further study to characterize fully the cooperation of these two kinases with integrins and Rho GTPases in the motility of cancer cells, cell–cell adhesion and cell–ECM interaction.

Determination of the mechanism maintaining tumour stemness is one of the most important challenges in current oncology and might also involve collaboration of RSK and EphA2. Binda *et al.*^7^ showed that EphA2 drives self-renewal and tumorigenicity in cancer stem cells derived from human glioblastoma, in which a strong signal for pS-EphA2 was detected in stem-like tumour propagating cells, but not in differentiated astrocytoma and neuroblastoma, in an ephrin-A ligand-independent manner. In addition, infiltrative invasion of glioma stem cells expressing EphA2 *in vivo*, which is completely independent of ephrin-A1, A3 and A4 ligands, was disrupted by S897A mutation^43^. Stratford *et al.*^44^ reported that targeting RSK2 with siRNA or small molecule inhibitors eliminates tumour-initiating cells in triple-negative breast cancers. Combining these reports with our results establishes the new idea that the RSK–EphA2 axis might have an important function in the maintenance of cancer stem properties; therefore, detailed study is needed to understand fully their roles in tumour progression.

EphA2 expression has been considered to be associated with poorer clinical outcomes in lung cancer patients^33^,^45^,^46^. Brannan *et al.*, for example, reported that EphA2 expression was positively correlated with activated EGFR, KRAS mutation, smoking history, poor prognosis, early recurrence and metastasis^33^,^45^. Here, we demonstrated that RSK-mediated EphA2 phosphorylation at Ser-897 is involved in poor patient survival, especially in smokers. It is well known that smoking is frequently associated with *KRAS* mutation^47^. In addition, KRAS activation of the ERK pathway induces EphA2 expression in cultured cancer cells *in vitro*^33^. Considering these observations together with our results, KRAS-mediated activation of the ERK–RSK pathway regulates not only EphA2 protein expression but also the phosphorylation of EphA2, which consequently results in high level expression of pS-EphA2 leading to cancer metastasis. In addition to *KRAS* mutation, we demonstrated that *EGFR* mutation and *ALK* fusion, major causes of NSCLCs, are other potential mechanisms to activate RSK in human NSCLCs (Fig. 6). In contrast, the regulation of EphA2 overexpression is still largely unknown; therefore, further detailed study is needed to understand fully the molecular mechanisms behind the high expression of pS-EphA2 in cancer cells.

The role of pS-EphA2 has also been demonstrated in glioblastoma multiforme, in which a high level of pS-EphA2 was only detected in grade IV human astrocytomas^18^. These results suggest that the RSK–EphA2 axis is a common pathophysiological signature for human cancers. In the present study, we demonstrated that BRAF-V600E evoked RSK-mediated EphA2 phosphorylation in melanoma cells. It has been demonstrated that both RSK and EphA2 play critical oncogenic and cancer progressive roles in melanoma^48^,^49^. Moreover, it has become evident that ligand-independent EphA2 signalling is a mediator of vemrafenib resistance^50^,^51^. Recent clinical trials clarified that combined MEK (trametinib) and BRAF (dabrafenib) inhibition is effective molecular-targeted therapy for melanoma patients harbouring BRAF mutation, suggesting that the RSK–EphA2 pathway inhibition affects the therapeutic efficacy^52^. As shown in Supplementary Fig. 7, treatment of trametinib resulted in effective downregulation of the RSK–EphA2 pathway; therefore, it is one possible strategy for intervention of phosphorylation of EphA2 for the treatment of cancers in a clinical context.

In summary, we have demonstrated the previously unknown connection of RSK to EphA2, and this pathway is involved in the malignant progression of cancer cells, such as migration and invasion. In addition to EphA2, many other RTKs are overexpressed in tumour tissues and involved in oncogenesis and acquired resistance to molecular-targeted agents. Therefore, study on ligand-controlled and ligand-independent serine and threonine phosphorylation is the next challenge to elucidate new unknown functions of RTKs, to illustrate cancer pathology and to identify new molecular targets for pharmacological interventions.

## Methods

### Antibodies and reagents

The phospho-specific antibodies against EphA2 (Ser-897; #6347 and Tyr-588; #12677), Akt (Ser-473; #9271), RSK1 (Ser-380; cross-reacting with RSK2 Ser-386; #11989), ERK (Thr-202/Tyr-204; #9101) and EGFR (Thr-669; #3056, Ser-1046/7; #2238 and Tyr-1068; #2236) were purchased from Cell Signaling Technology (Danvers, MA, USA). Antibodies against total EphA2 (C-20; sc-924), RSK1 (C-21; sc-231), RSK2 (C-19; sc-1430), EGFR (1005; sc-03), TAK1 (M-579; sc-7162), β-actin (I-19; sc-1616) and α-tubulin (B-7; sc-5286) were obtained from Santa Cruz Biotechnology (Santa Cruz, CA, USA). Recombinant human TNF-α, ephrin-A1-Fc chimera and EGF were obtained from R&D Systems (Minneapolis, MN, USA); recombinant human active GST-EphA2, GST-RSK1 and GST-RSK2 protein were from Carna Biosciences (Kobe, Japan); anti-EGFR monoclonal antibody (clone LA1; 05-101) was from Millipore (Billerica, MA, USA); Phos-tag ligand and TPA were from Wako Pure Chemical Industries (Osaka, Japan); LY294002, SB203580 and U0126 were from Merck Biosciences (Darmstadt, Germany); MK-2206 was from Active Biochemicals (Wan Chai, Hong Kong); BI-D1870 and crizotinib were from BioVision (Milpitas, CA, USA); gefitinib was from Cayman Chemical (Ann Arbor, MI, USA); and vemurafenib was from LC Laboratories (Woburn, MA, USA). All of the chemical inhibitors were dissolved in Me_2_SO, and the final concentration of Me_2_SO was <0.1%.

### Cell cultures

HeLa, HEK293, MDA-MB-231, Panc-1 and A375 cells were obtained from the American Type Culture Collection (ATCC, Rockville, TX, USA) and maintained in Dulbecco's modified Eagle's medium (high-glucose condition; Life Technologies Corporation, Carlsbad, CA, USA) supplemented with 10% fetal calf serum, 100 U ml^−1^ penicillin and 100 μg ml^−1^ streptomycin (Meiji Seika Pharma, Tokyo, Japan) at 37 °C in 5% CO_2_. DLD-1 (kindly gifted by Dr K. Tsukada, University of Toyama, Toyama, Japan), A2058 (ATCC), SK-MEL-28, SK-MEL-2, UACC62, UACC257 (kindly gifted by Dr D. E. Fisher, Massachusetts General Hospital, Boston, MA, USA), PC-9 (kindly gifted by Dr K. Kiura, Okayama University, Okayama, Japan), HCC827 (ATCC), HCC4006 (kindly gifted by Dr A.F. Gazdar, University of Texas Southwestern Medical Center, Dallas, TX, USA), NCI-H1650 (ATCC), H2228 (ATCC) and A549 (ATCC) cells were cultured in RPMI 1640 medium (Life Technologies Corporation) supplemented with 10% fetal calf serum, 100 U ml^−1^ penicillin and 100 μg ml^−1^ streptomycin at 37 °C in 5% CO_2_. T98G and U-87 MG cells kindly gifted by Dr M. Takeya (Kumamoto University, Kumamoto, Japan) and T. Imanaka (University of Toyama), respectively were maintained in Eagle's MEM (Nissui, Tokyo, Japan) supplemented with 10% fetal calf serum and 2 mM L-glutamine (Life Technologies Corporation) at 37 °C in 5% CO_2_. Stably Luc- and TAK1-knocked down HeLa cells were as described previously^12^.

### Transfection of plasmid DNAs

Expression vectors for human wild-type (WT) and kinase-dead (KD) mutant EphA2 were provided by Dr Haruhiko Sugimura (Hamamatsu University School of Medicine, Hamamatsu, Japan) and were as described previously^53^,^54^. The expression vector for human RSK1 was provided by Dr Yoshikazu Sugimoto (Keio University, Tokyo, Japan) and was as described previously^55^. HEK293 and MDA-MB-231 cells were transfected using Lipofectamine 2000 or Lipofectamine LTX (Life Technologies Corporation) in accordance with the manufacturer's instructions. S897A(SA)-EphA2 or KDSA-EphA2 substitution mutations for WT-EphA2 or KD-EphA2 expression plasmids, respectively, and constitutively active (CA-RSK1 (Y702A)) and kinase-dead (KD-RSK1 (K94R/K447R)) mutations for RSK1 expression plasmid were generated by PCR with PrimeSTAR DNA polymerase (Takara Bio, Shiga, Japan).

### RNA interference

Small interfering RNAs (siRNAs) were synthesized at Life Technologies Corporation (Stealth RNA interference) or Hokkaido System Science Co., Ltd. (Sapporo, Japan). The target sequences were as follows: 5′-CCAUGCUGGCAGGAUAUACUCCAUU-3′ (RSK1), 5′-GGGAGGAGAUUUGUUUACACGCUUA-3′ (RSK2), 5′-UGGAGUCCAUCAAGAUGCAGCAGUA-3′ (EphA2), 5′-UGGCUUAUCUUACACUGGA-3′ (TAK1) and 5′-UAAUGUACUGCGCGUGGAGAGGAA-3′ (negative control). HeLa and MDA-MB-231 cells were transfected with siRNAs at a final concentration of 20 to 100 nM using Lipofectamine Reagent or Lipofectamine LTX (Life Technologies Corporation) in accordance with the manufacturer's instructions, respectively.

### Immunoblotting

Whole-cell lysates were prepared with lysis buffer (25 mM HEPES (pH 7.7), 0.3 M NaCl, 1.5 mM MgCl_2_, 0.2 mM EDTA, 0.1% Triton X-100, 20 mM β-glycerophosphate, 1 mM sodium orthovanadate, 1 mM phenylmethylsulfonyl fluoride, 1 mM dithiothreitol, 10 μg ml^−1^ aprotinin and 10 μg ml^−1^ leupeptin). The lysates were mixed with the same volume of SDS–PAGE sample buffer (100 mM Tris-HCl (pH 6.8), 2.0% SDS, 70 mM DTT, 10% glycerol and 0.10% bromophenol blue) and heated at 95 °C for 5 min. Samples were resolved using 6.5, 7.5 or 10% SDS–PAGE gel and transferred to an Immobilon-P nylon membrane (Millipore, Billerica, MA, USA). The membrane was treated with BlockAce (Dainippon Sumitomo Pharmaceutical Co., Ltd., Osaka, Japan) and probed with primary antibodies (diluted 1:1,000, except for phospho-ERK, RSK1, β-actin and α-tubulin, which were diluted 1:4,000). The antibodies were detected using horseradish peroxidase-conjugated anti-rabbit (P0448; diluted 1:2,000), anti-mouse (P0260; diluted 1:2,000) and anti-goat IgG (P0449; diluted 1:2,000; DAKO, Glostrup, Denmark) and visualized with the ECL system (GE Healthcare Bioscience, Piscataway, NJ, USA). Some antibody reactions were carried out in Can Get Signal solution (TOYOBO, Tokyo, Japan) or PBS containing 0.1% Tween 20 (Wako Pure Chemical Industries). Uncropped scans of the blots were supplied in Supplementary Fig. 11. Analysis was carried out at least three times and representative results are shown.

### Zn^2+^-Phos-tag SDS–PAGE

For phos-tag gel, whole-cell lysates were prepared with RIPA buffer (50 mM Tris-HCl (pH 7.4), 0.15 M NaCl, 0.25% sodium deoxycholate, 1.0% Nonidet P-40, 1.0 mM EDTA, 20 mM β-glycerophosphate, 1 mM sodium orthovanadate, 1 mM phenylmethylsulfonyl fluoride, 1 mM dithiothreitol, 10 μg ml^−1^ aprotinin and 10 μg ml^−1^ leupeptin). Each sample was mixed with a half volume of SDS–PAGE sample buffer (195 mM Tris-HCl (pH 6.8), 3.0% SDS, 15% 2-mercaptoethanol, 30% glycerol and 0.10% bromophenol blue) and heated at 95 °C for 5 min. The procedures for Zn^2+^-Phos-tag SDS–PAGE were as described previously^20^. In brief, the acrylamide pendant Phos-tag ligand and two equivalents of ZnCl_2_ were added to the separating gel before polymerization. The running buffer consisted of 100 mM Tris and 100 mM MOPS containing 0.10% SDS and 5.0 mM sodium bisulfite. After Zn^2+^-Phos-tag SDS–PAGE, the gel was soaked in a solution containing 25 mM Tris, 192 mM glycine, 10% MeOH and 1.0 mM EDTA for 20 min and then soaked in a solution containing 25 mM Tris, 192 mM glycine and 10% MeOH for 20 min. Gel transfer, blocking, antibody reaction and detection were by the same methods as for immunoblotting.

### *In vitro* kinase assay

Recombinant human GST-EphA2 (70 ng) was reacted with recombinant human active GST-RSK1 (100 ng) or RSK2 (100 ng) at 30 °C for 30 min in 30 μl of reaction buffer containing 20 mM HEPES (pH 7.6), 20 mM MgCl_2_, 0.2 mM ATP, 2 mM DTT, 20 mM β-glycerophosphate and 0.1 mM sodium orthovanadate. After stopping the reaction by adding 30 μl of SDS–PAGE sample buffer, immunoblotting was performed as described above.

### Fluorescence microscopy

Cells seeded on a coverslip (Thermo Fisher Scientific, Waltham, MA, USA) were fixed with 2% paraformaldehyde (Muto Pure Chemicals, Tokyo, Japan) for 20 min and permeabilized with 0.5% Triton X-100 (Wako Pure Chemical Industries) for 5 min. The coverslips were overlaid with primary antibody diluted 1:100 for EphA2 or at 5 μg ml^−1^ for EGFR in PBS with 0.5% BSA (Nakarai tesque, Kyoto, Japan) and incubated for 2 h, then with Alexa Fluor 488-conjugated goat anti-rabbit IgG antibody (A11008, diluted 1:500; Life Technologies Corporation) in PBS with 0.5% BSA for 1 h, and finally with Rhodamine Phalloidin ‘Life Technologies Corporation' for 30 min. After being washed with PBS, the coverslips were inverted onto a slide with SlowFade Gold Antifade Reagent with DAPI (Life Technologies Corporation). Fluorescence was analysed by LSM700 confocal microscopy (Zeiss, Oberkochen, Germany). The number of cells harbouring and losing lamellipodia in the scratched area was counted. Image capture and quantification was performed masked to experimental conditions. Analysis was carried out at least three times and representative results are shown.

### Scratch assay

The cells were grown as a monolayer and scratched using a pipette tip through the monolayer. The cells were washed to remove cellular debris and allowed to migrate for 48 h. The number of migrated cells in the scratched area was counted. Analysis was carried out at least three times and representative results are shown. Quantification was performed masked to experimental conditions.

### Immunohistochemistry

A multi-cancer tissue microarray and a lung cancer tissue microarray, which consist of 1,010 cores from 13 organ cancer tissues (lung, breast, thyroid, liver, colon, stomach, prostate, ovary, uterine corpus, kidney, pancreas, biliary tract and urothelial cancers) and 353 cores, respectively, were obtained from Toyama University Hospital (Toyama, Japan). Detailed clinical and pathologic information, including patient demographics, smoking history and overall survival, was available for most patients in the lung cancer tissue microarray. The tumour specimens with *EGFR* mutations were obtained from NSCLC patients, all of whom provided written informed consent, at Kanazawa University Hospital (Kanazawa, Japan). This study was approved by the Institutional Review Boards of each institute.

Immunohistochemistry was performed at Pathology Institute Corporation (Toyama, Japan). The sections were deparaffinized and subjected to heat-induced antigen retrieval using a water bath or a pressure chamber, blocking of the endogenous peroxidase with 3% hydrogen peroxide for 10 min and equilibration. Then, the sections were incubated with anti-phospho-Ser-897-EphA2 antibody (1:100) and anti-phospho-RSK1 antibody (1:50) at room temperature overnight. After extensive washing, the sections were incubated with EnVision+Dual Link System-HRP (DAKO) for 30 min, visualized with DAB and counterstained with haematoxylin, dehydrated, cleared and mounted with resinous mounting medium. Cytoplasmic and/or cell membrane staining for phospho-Ser-897-EphA2 and nuclear and/or cytoplasmic staining for phospho-RSK1 were considered to indicate positivity. Image capture and evaluation was performed in a masked manner.

### Statistical analysis

Statistically significant differences within each set of categorical data were determined using two-sided Fisher's exact tests. Overall survival was calculated using the Kaplan–Meier method and compared by two-sided log-rank statistics. Statistical analyses were performed using JMP software version 11 (SAS Institute Japan, Tokyo, Japan). Probability values of *P*<0.05 were considered statistically significant.

## Additional information

**How to cite this article:** Zhou, Y. *et al.* Crucial roles of RSK in cell motility by catalysing serine phosphorylation of EphA2. *Nat. Commun.* 6:7679 doi: 10.1038/ncomms8679 (2015).

## Supplementary Material

Supplementary Information Supplementary Figures 1-11, Supplementary Table 1, Supplementary Methods and Supplementary References.
