# Supplementary material for: Crucial roles of RSK in cell motility by catalysing serine phosphorylation of EphA2
Source: Nat Commun. 2015 Jul 9;6:7679. doi: 10.1038/ncomms8679 (PMC4510653; doi:10.1038/ncomms8679)
Supplement: Supplementary Information — Supplementary Figures 1-11, Supplementary Table 1, Supplementary Methods and Supplementary References. [file ncomms8679-s1.pdf]

Supplementary Figure 1

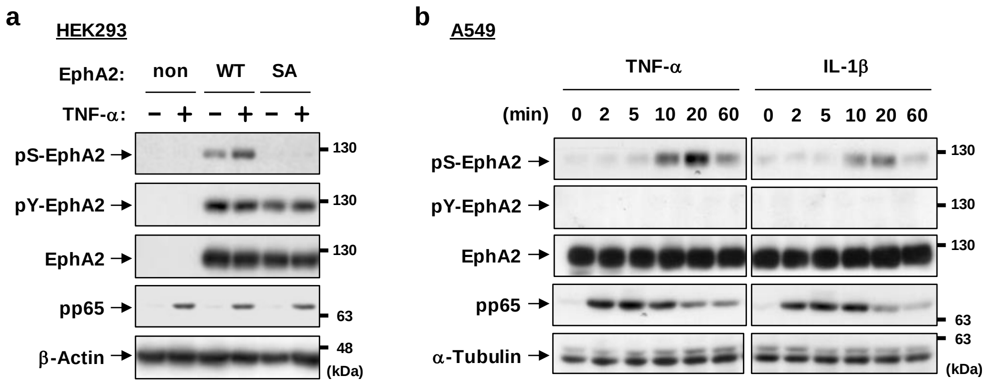

Supplementary Figure 1. Phosphorylation of EphA2 at Ser-897 is induced by an inflammatory signal. (a) HEK293 cells were transfected with an expression vector for EphA2 and its substitution mutant. At 24 hrs post-transfection, cells were treated with TNF-α for 20 min. Whole cell lysates were immunoblotted with anti-pS-EphA2, pY-EphA2, EphA2, pp65 and β-actin antibodies. (b) Whole cell lysates from A549 cells treated with TNF-α (20 ng ml^{-1}) or IL-1β (10 ng ml^{-1}) were electrophoresed and probed with primary antibodies against pS-EphA2, pY-EphA2, EphA2, pp65 and α-tubulin.

Supplementary Figure 2

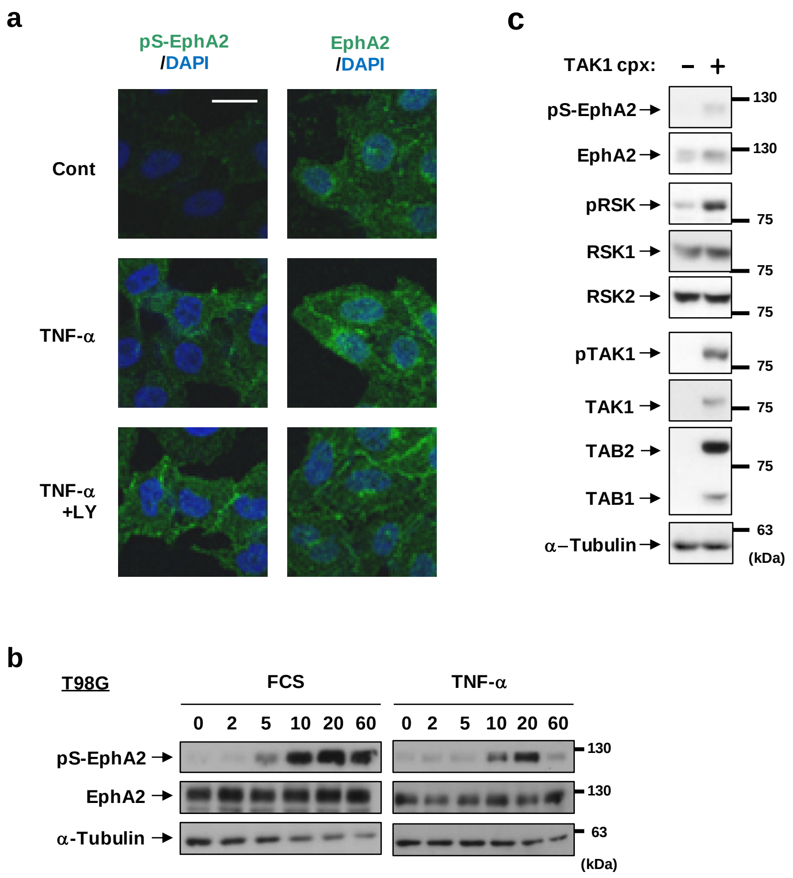

Supplementary Figure 2. TAK1 promotes the phosphorylation of EphA2. (a) HeLa cells were pre-treated with LY294002 for 30 min and then stimulated with TNF-α for 20 min. After fixation and permeabilization, cells were stained with pS-EphA2 or EphA2 antibodies. Scale bar represents 20 µm. Shown are representative images from three independent experiments. (b) Whole cell lysates from T98G cells treated with FCS or TNF-α for the indicated period were electrophoresed and probed with primary antibodies against pS-EphA2, EphA2 and α-tubulin. (c) HeLa cells were transfected with expression vectors for TAK1, TAB1 and TAB2. At 24 hrs post-transfection, whole cell lysates were immunoblotted with anti-pS-EphA2, EphA2, pRSK, RSK1, RSK2, pTAK1, TAK1, TAB1, TAB2 and α-tubulin antibodies.

Supplementary Figure 3

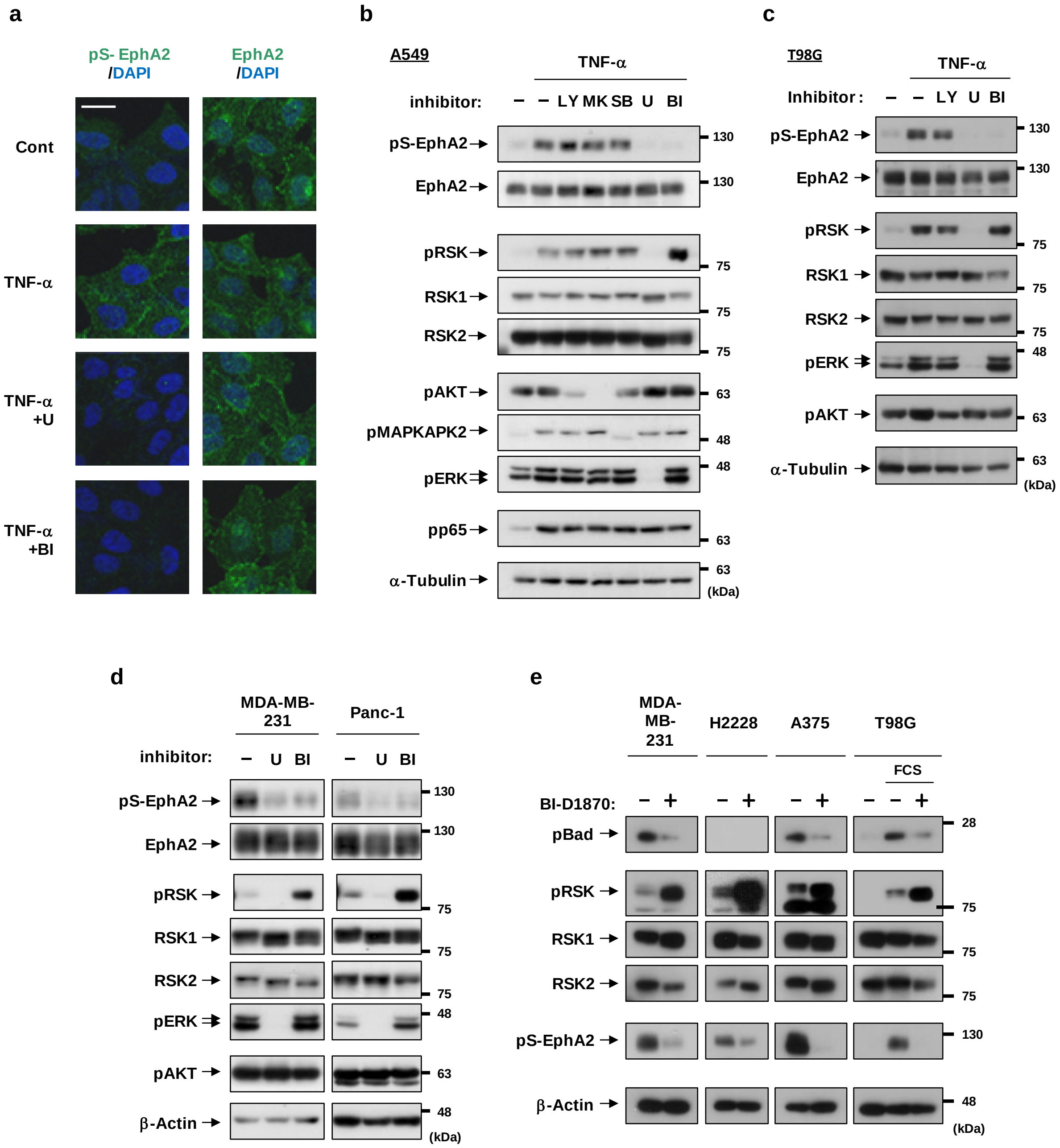

Supplementary Figure 3 (continued)

Supplementary Figure 3. Phosphorylation of EphA2 at Ser-897 is induced by the ERK-RSK pathway. (a) HeLa cells were pre-treated with U0126 or BI-D1870 for 30 min and then stimulated with TNF-α for 20 min. After fixation and permeabilization, cells were stained with pS-EphA2 or EphA2 antibodies. Scale bar represents 20 μm. Shown are representative images from three independent experiments. (b) A549 cells were pre-treated with LY294002, MK2206, SB203580, U0126 or BI-D1870 for 30 min, and then stimulated with TNF-α for 20 min. Whole cell lysates were immunoblotted with anti-pS-EphA2, EphA2, pRSK, RSK1, RSK2, TAB2, pAkt, pMAPKAPK2, pERK, pp65 and α-tubulin antibodies. (c, d) T98G cells were pre-treated with LY294002, U0126 or BI-D1870 for 30 min and then stimulated with TNF-α (c). MDA-MB-231 and Panc-1 cells were treated with U0126 or BI-D1870 for 30 min (d). Whole cell lysates were electrophoresed and probed with primary antibodies against pS-EphA2, EphA2, pRSK, RSK1, RSK2, pERK, pAkt and α-tubulin. (e) MDA-MB-231, H2228 and A549 cells were treated with BI-D1870 for 30 min. T98G cells starved in FCS-free medium for 24 hr were treated with BI-D1870 for 30 min, and then treated with 10% FCS for 10 min. Whole cell lysates were immunoblotted with anti-pBad, pRSK, RSK1, RSK2, pS-EphA2 and β-actin antibodies.

Supplementary Figure 4

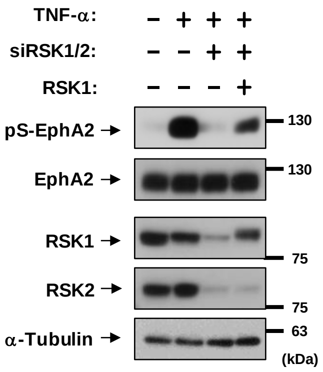

Supplementary Figure 4. pS-EphA2 is rescued by RSK1 re-expression in RSK1/2 knockdown cells. HeLa cells were transfected with siRNAs against RSK1/2 or negative control. At 48 hrs post-transfection cells were transfected with an RSK1 expression vector or an empty vector. After 24 hrs, whole cell lysates were immunoblotted with anti-pS-EphA2, EphA2, RSK1, RSK2 and α-tubulin antibodies

Supplementary Figure 5

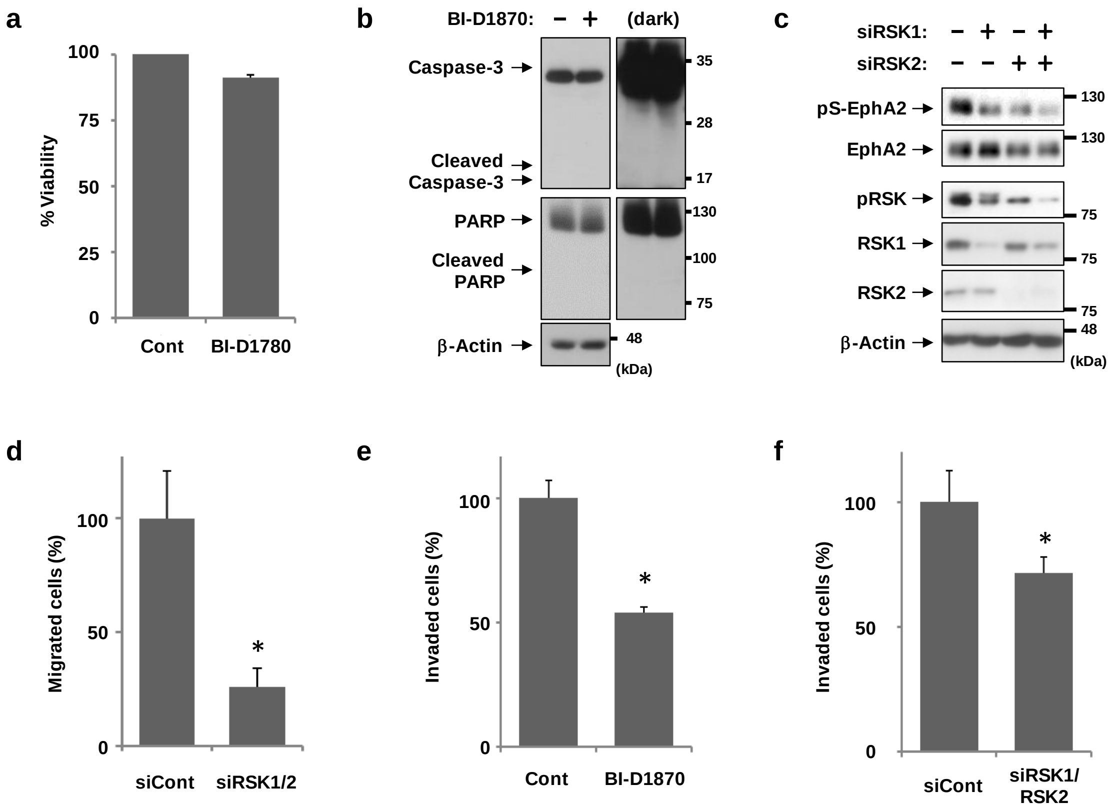

Supplementary Figure 5. Cell migration and invasion are controlled by the RSK-EphA2 axis. (a, b, e) MDA-MB-231 cells treated with BI-D1870 for 48 hrs were employed for the cell viability assay (a) and the Matrigel invasion assay (e). In addition, whole cell lysates were immunoblotted with anti-Caspase-3, PARP and β-Actin antibodies (b). (c, d, e) MDA-MB-231 cells were transfected with siRNAs against RSK1, RSK2 or negative control. At 72 hrs post-transfection, whole cell lysates were immunoblotted with anti-pS-EphA2, EphA2, pRSK, RSK1, RSK2 and β-actin antibodies (c). In addition, cells were scratched with a pipette tip for the scratch assay. Migrated cells were counted manually under a microscope 48 hrs after scratching (d). Moreover, cells were peeled off from the culture dish and used for the Matrigel invasion assay (f). All data are the means ± SD of at least three fields. Similar results were obtained in at least three independent experiments. *$P < 0.05$ by Student's *t*-test.

Supplementary Figure 6

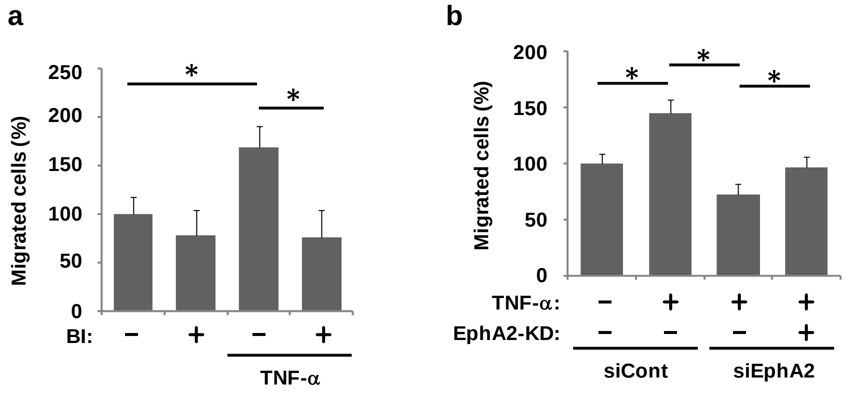

Supplementary Figure 6. TNF-α-induced migration is controlled by the RSK-EphA2 axis. A549 cells were pre-treated with BI-D1870 for 30 min (a) or transfected with siRNA against EphA2 or negative control and kinase-dead (KD) EphA2 expression plasmid (b), and then scratched with a pipette tip and stimulated with TNF-α. After 48 hrs of incubation, migrated cells were counted manually under a microscope. Data are the means ± SD of six fields. *$P < 0.05$ by ANOVA followed by Tukey-Kramer HSD test.

Supplementary Figure 7

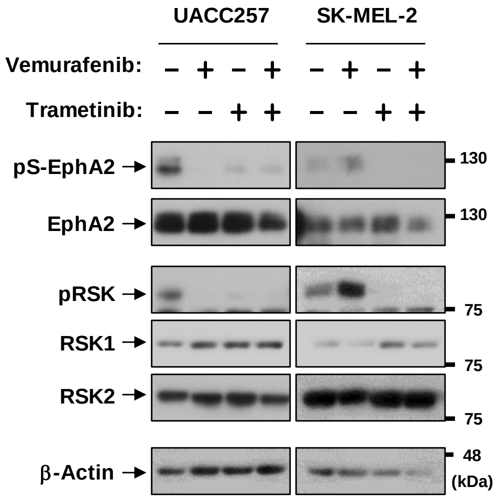

Supplementary Figure 7. Trametinib inhibited EphA2 phosphorylation. UACC257 and SK-MEL-2 were treated with vemurafenib (1 μM) and/or trametinib (30 nM) for 60 min. Whole cell lysates were immunoblotted with primary antibodies against pS-EphA2, EphA2, pRSK, RSK1, RSK2 and β-actin.

Supplementary Figure 8

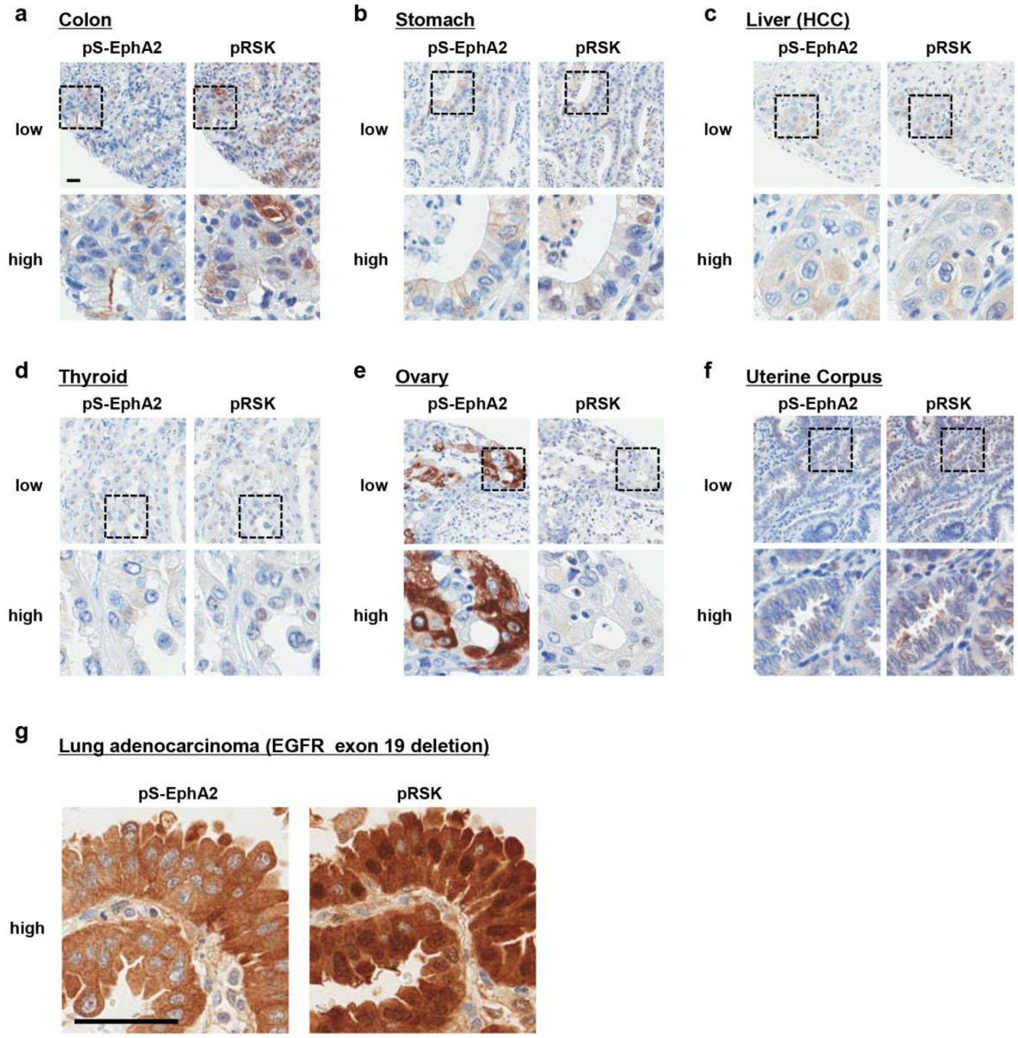

Supplementary Figure 8. pS-EphA2 and pRSK are colocalized in cancer patients' specimens. A multi-cancer tissue microarray, consisting of 1010 cores from 13 organ cancer tissues, was adopted for immunohistochemical staining using primary antibodies against pS-EphA2 and pRSK. Typical staining images of cancer tissues of colon (a), stomach (b), liver (c), thyroid (d), ovary (e), uterine corpus (f) and lung adenocarcinoma with EGFR exon 19 deletion (g) at low- and high-power magnification are shown. Scale bar represent 20 μm.

Supplementary Figure 9

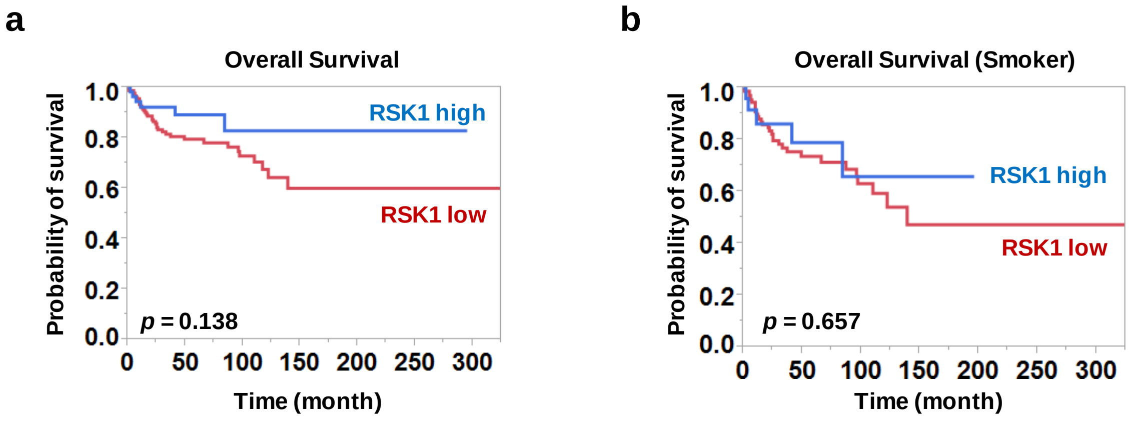

Supplementary Figure 9. RSK1 expression is not associated with the overall survival of lung cancer patients. Postoperative overall Kaplan-Meier survival curves of all lung cancer patients (a) or smoking patients (b) were compared according to RSK1 low (no staining and weak staining) or high (moderate and strong staining). *P*-values were calculated by the log-rank tests.

Supplementary Figure 10

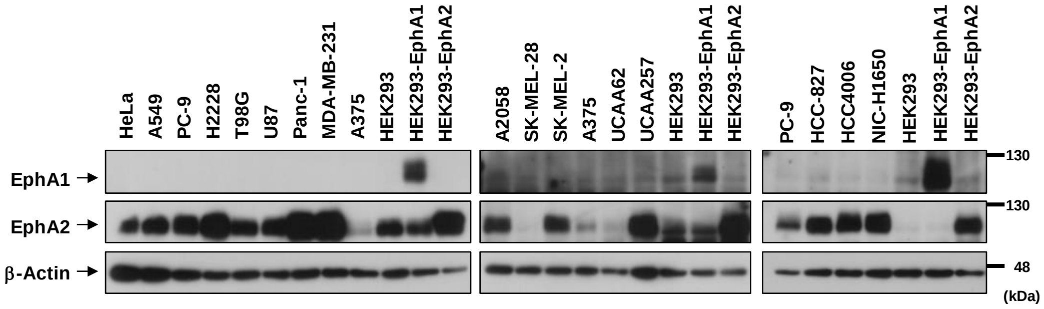

Supplementary Figure 10. EphA1 expression level in the cell lines used in this paper is very low. Whole cell lysates from indicated cells were immunoblotted with anti-EphA1, EphA2 and β-actin antibodies.

Supplementary Figure 11

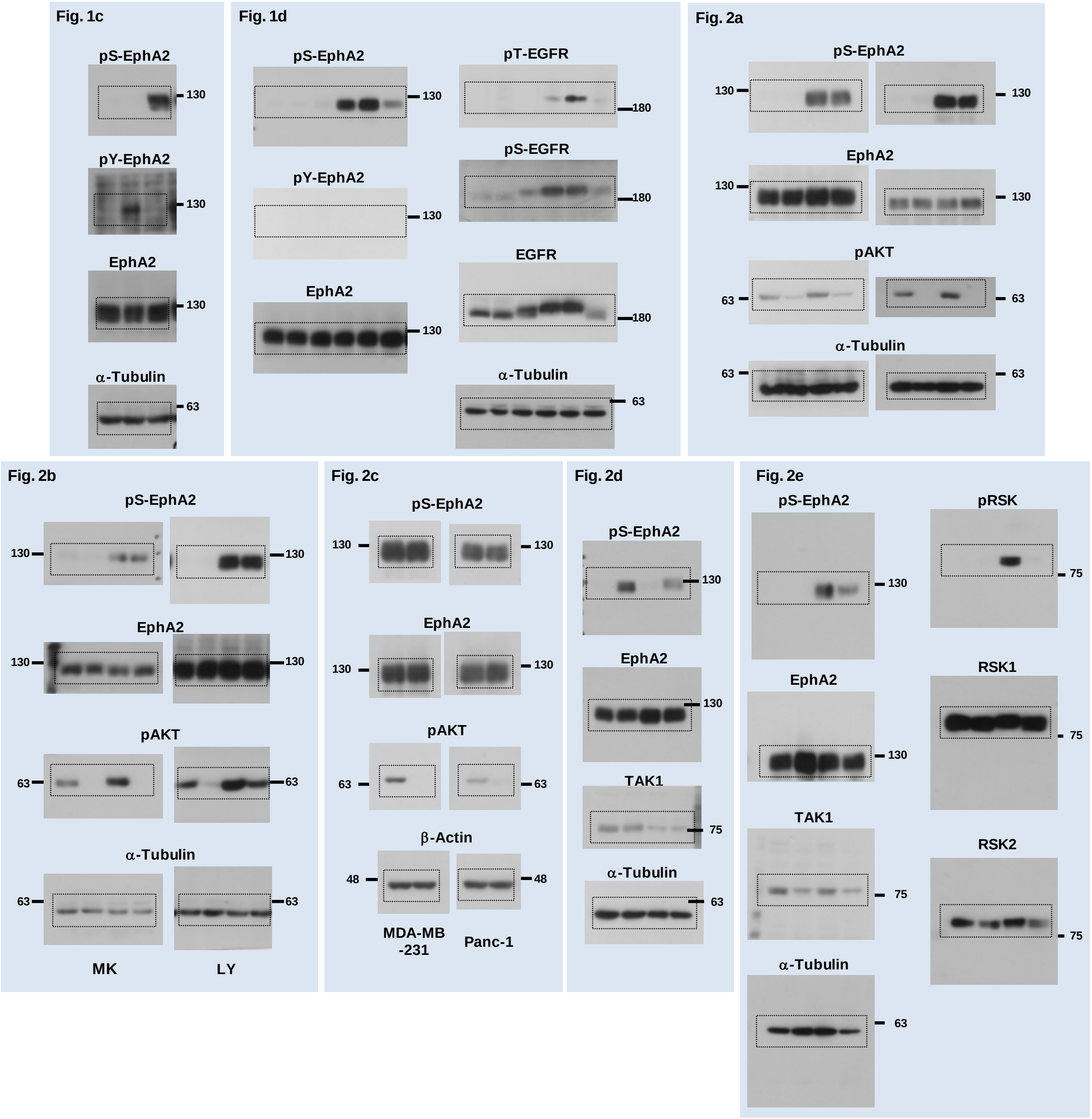

Supplementary Figure 11 (continued)

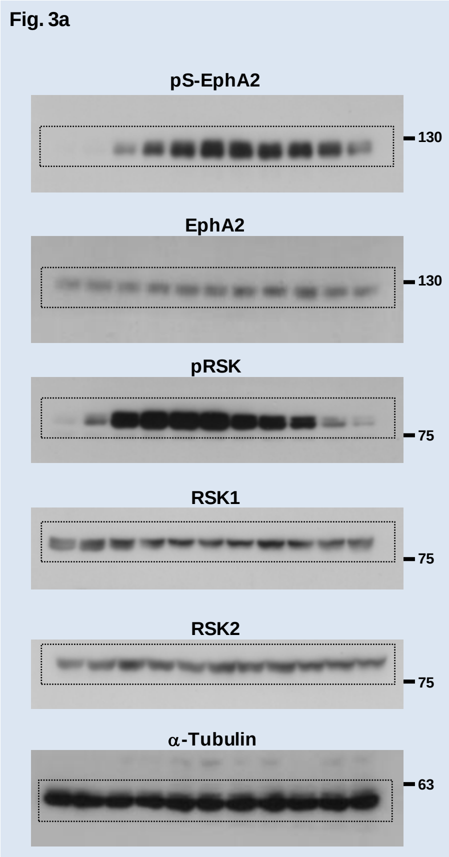

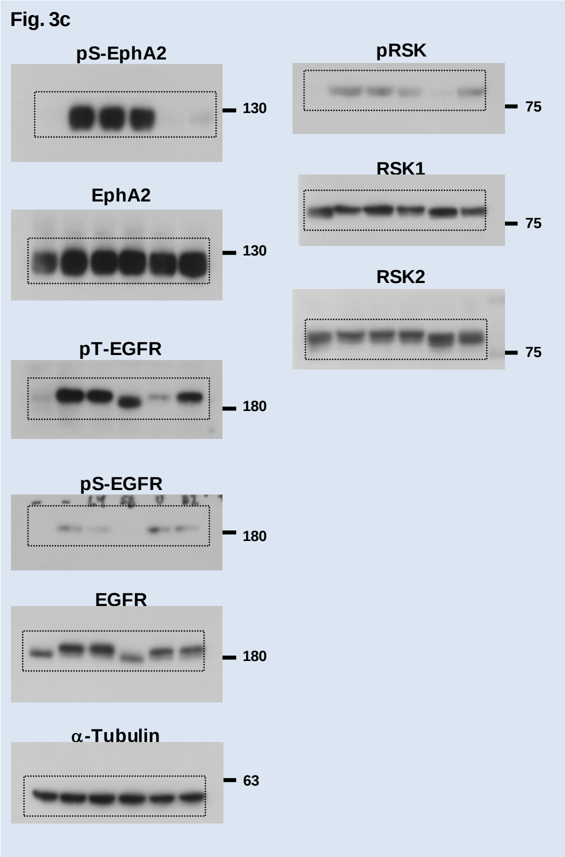

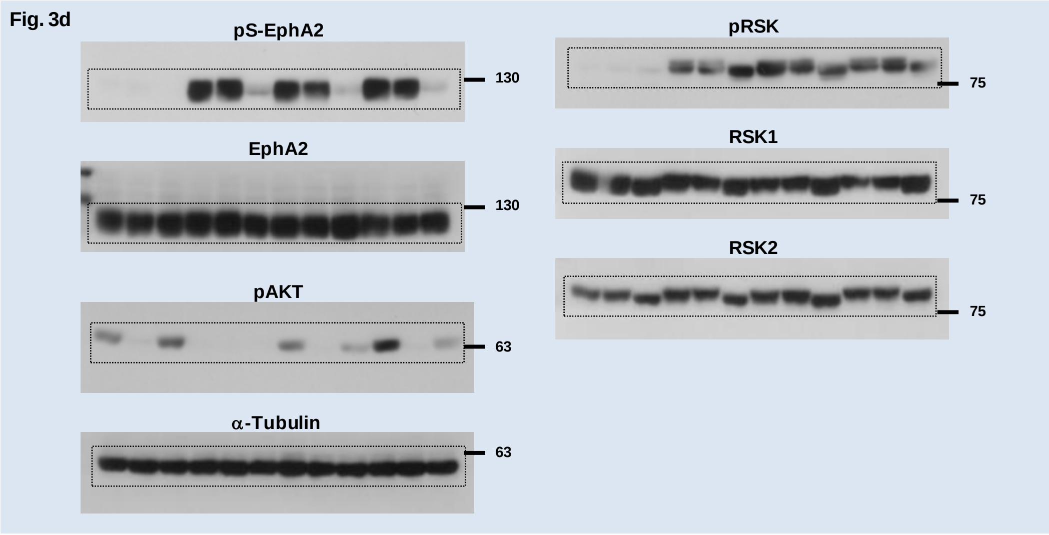

Supplementary Figure 11 (continued)

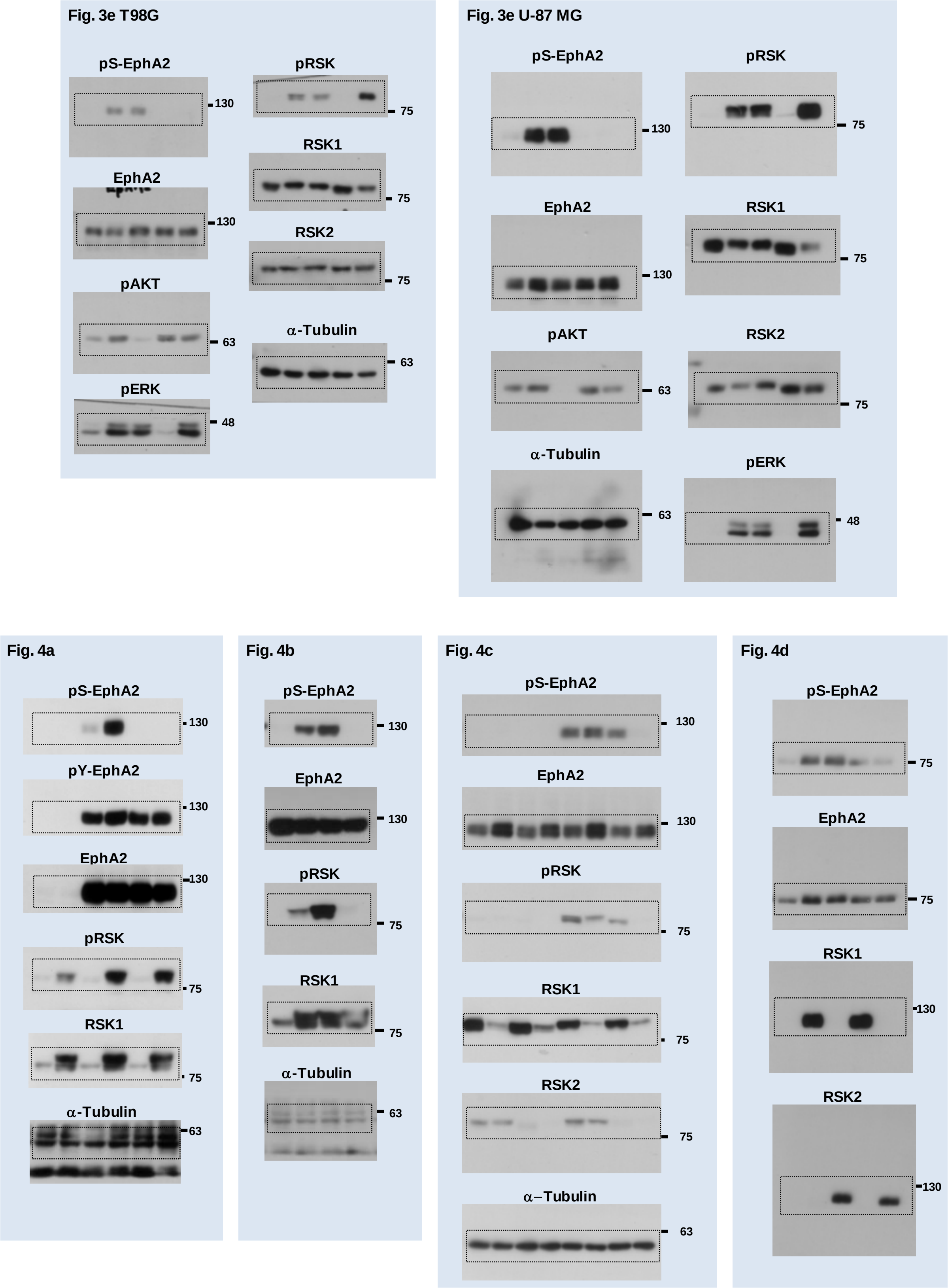

Supplementary Figure 11 (continued)

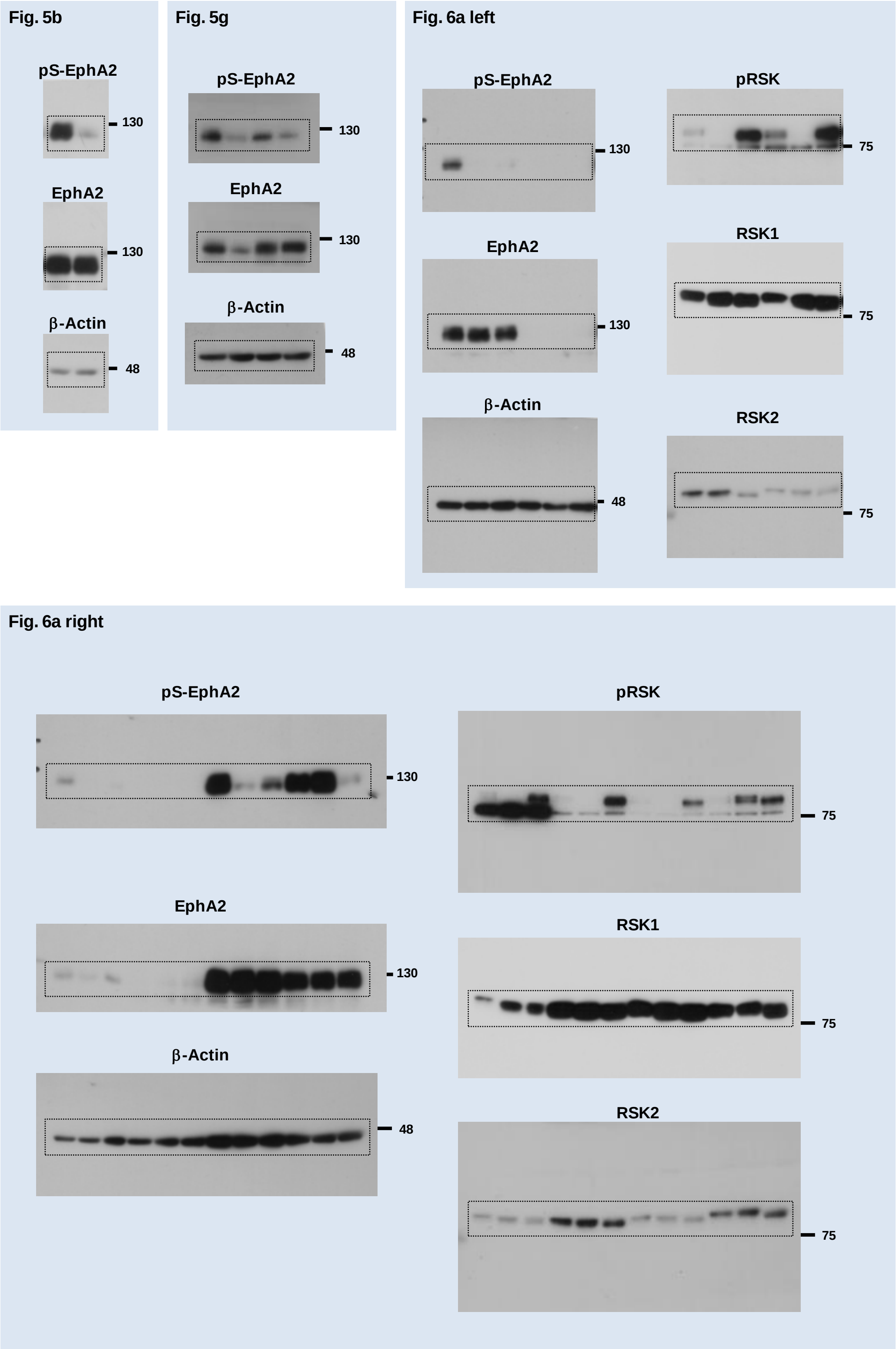

Supplementary Figure 11 (continued)

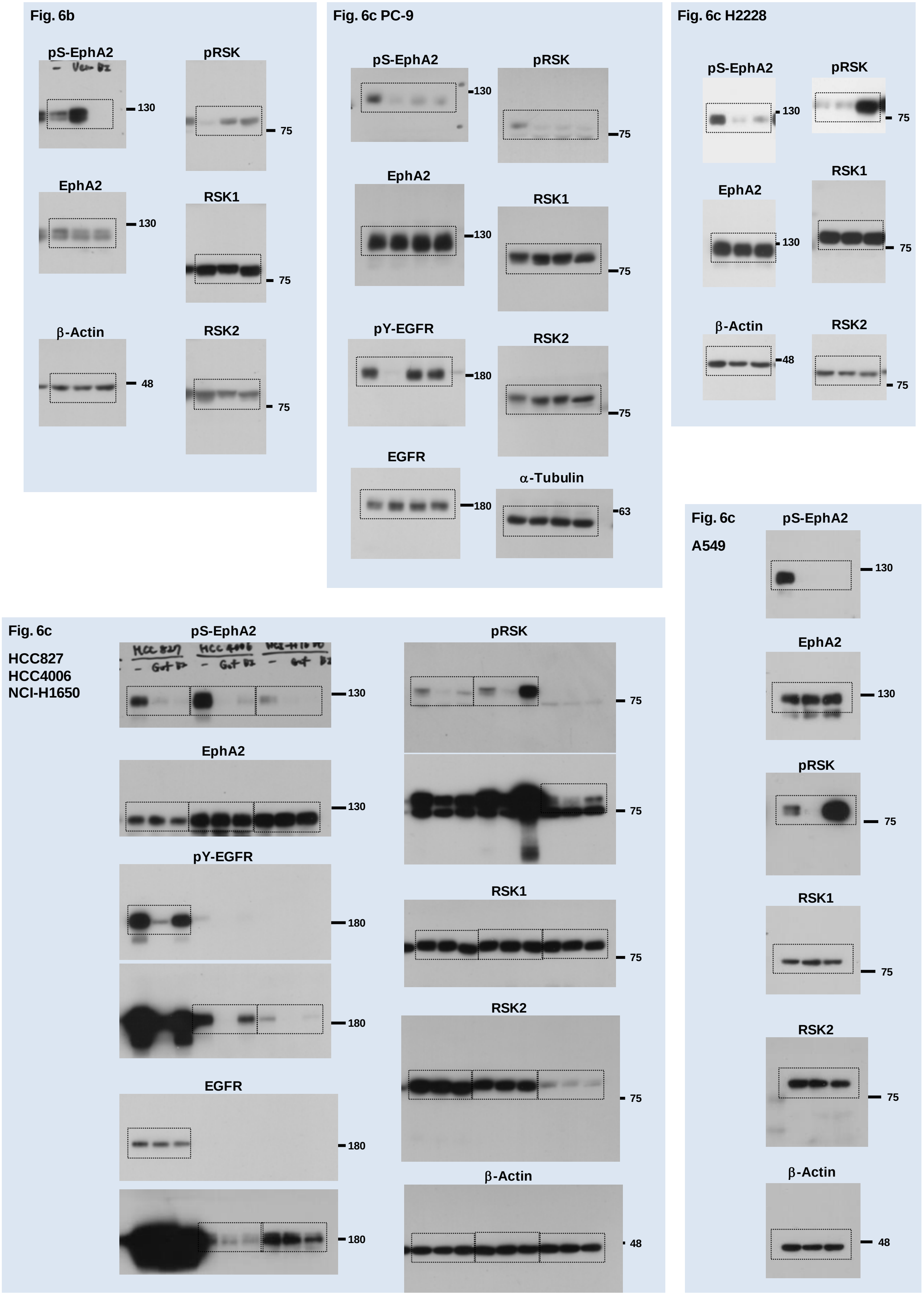

Supplementary Figure 11 (continued)

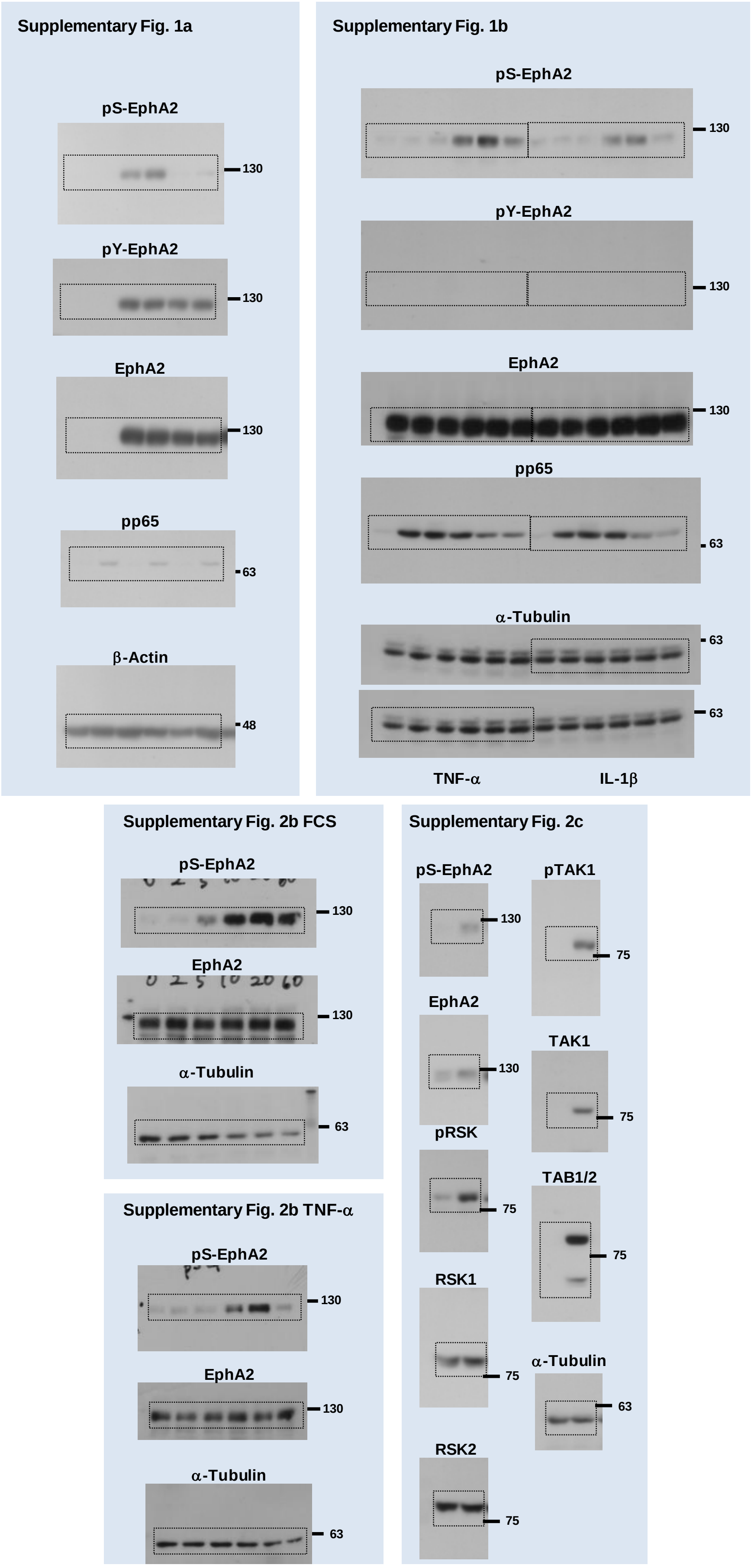

Supplementary Figure 11 (continued)

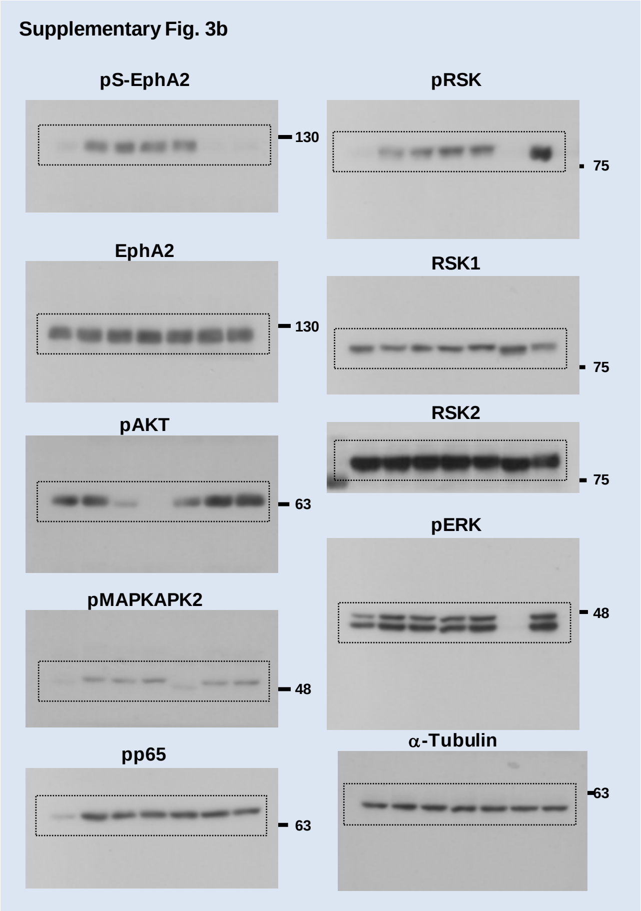

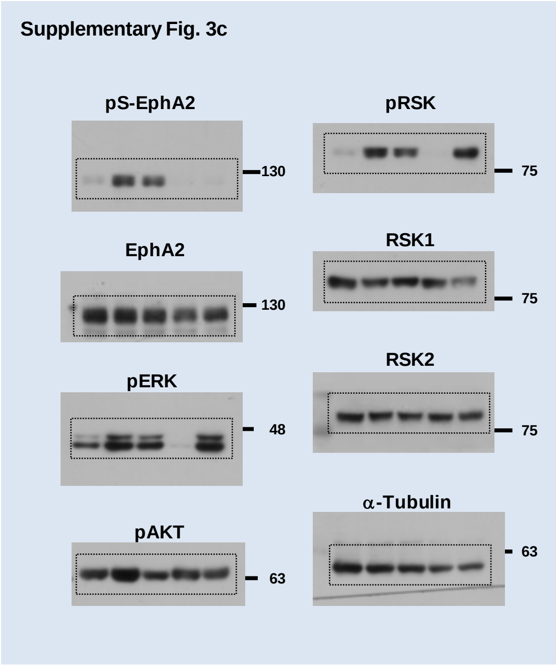

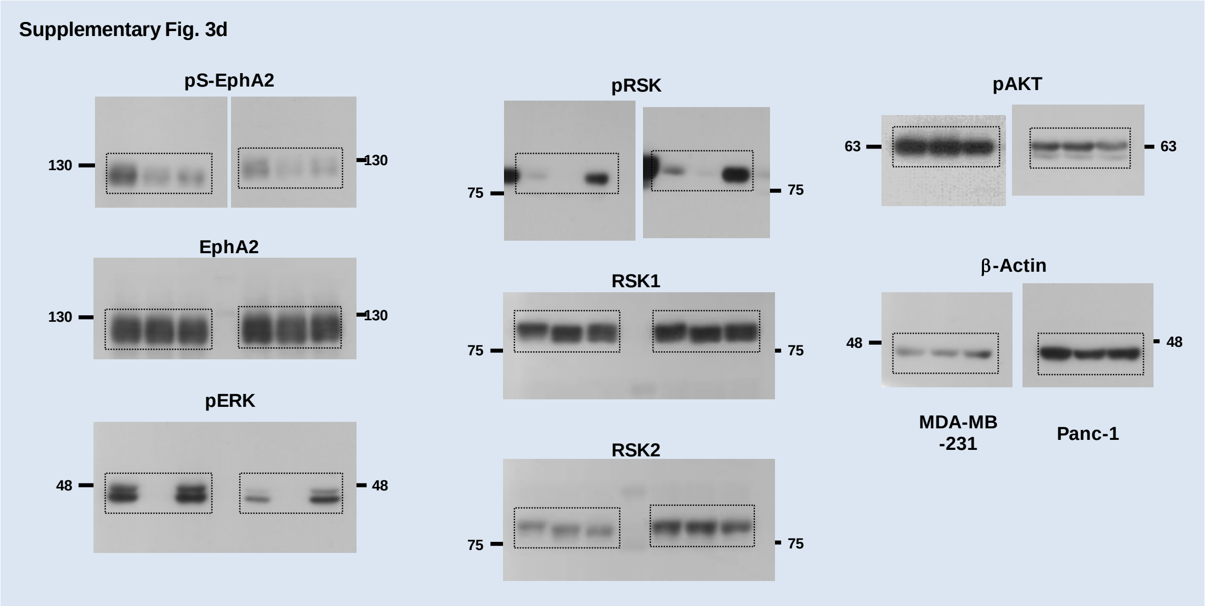

Supplementary Figure 11 (continued)

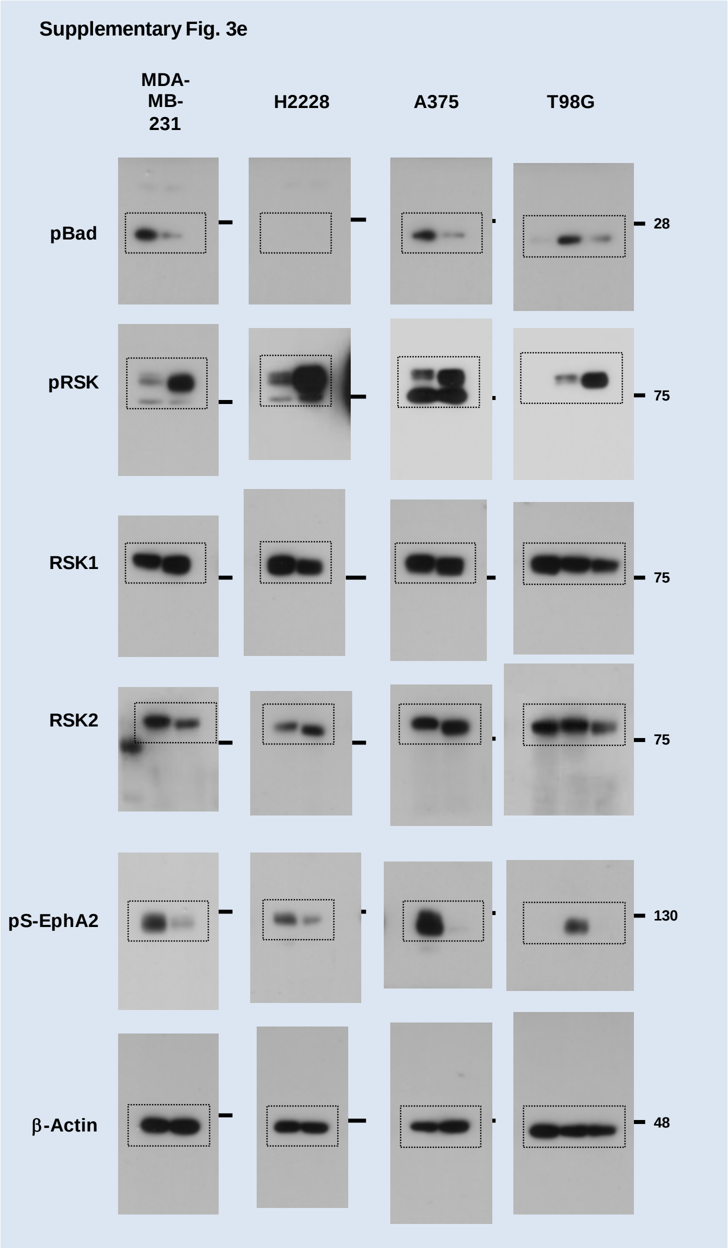

Supplementary Figure 11 (continued)

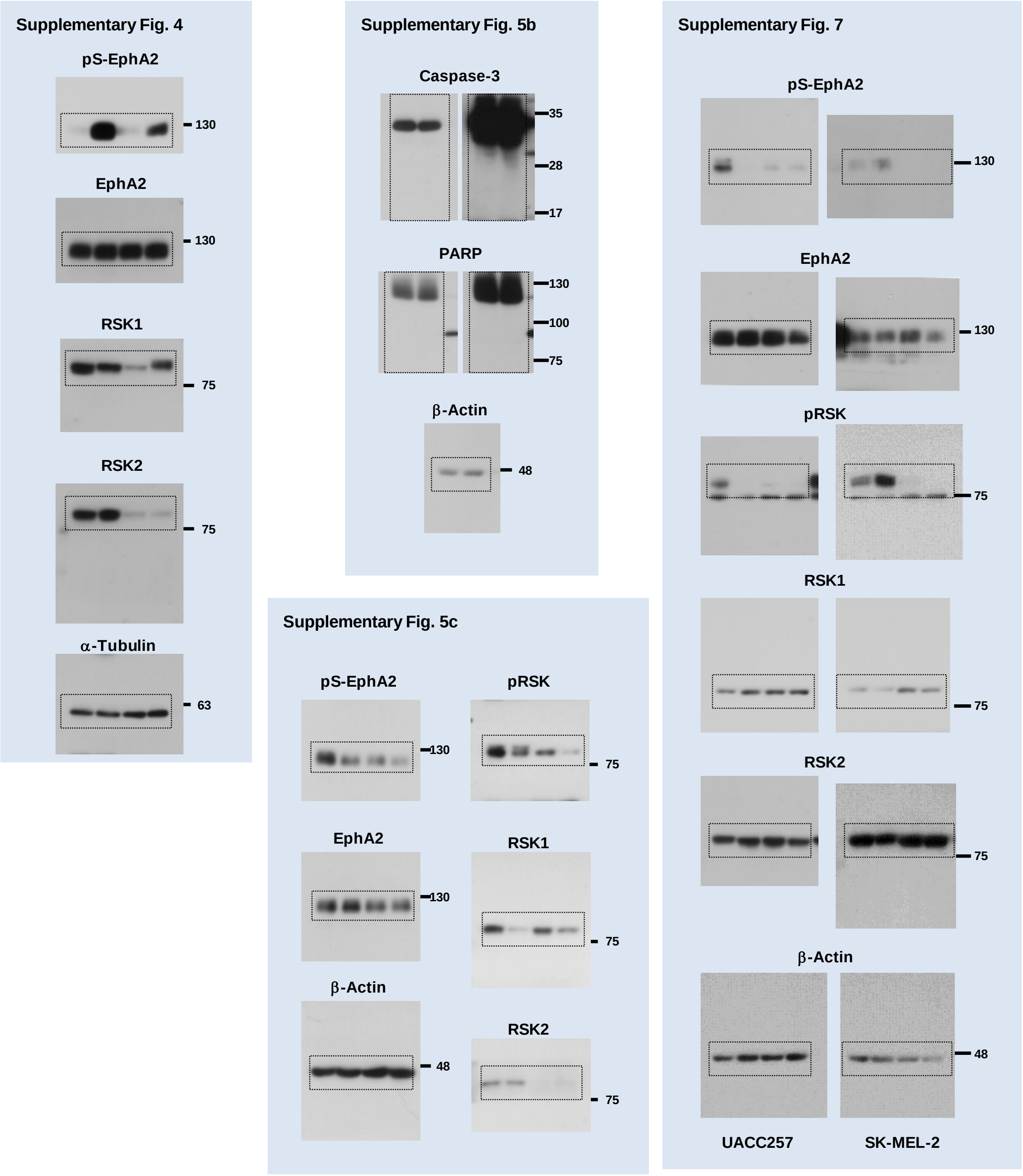

Supplementary Figure 11 (continued)

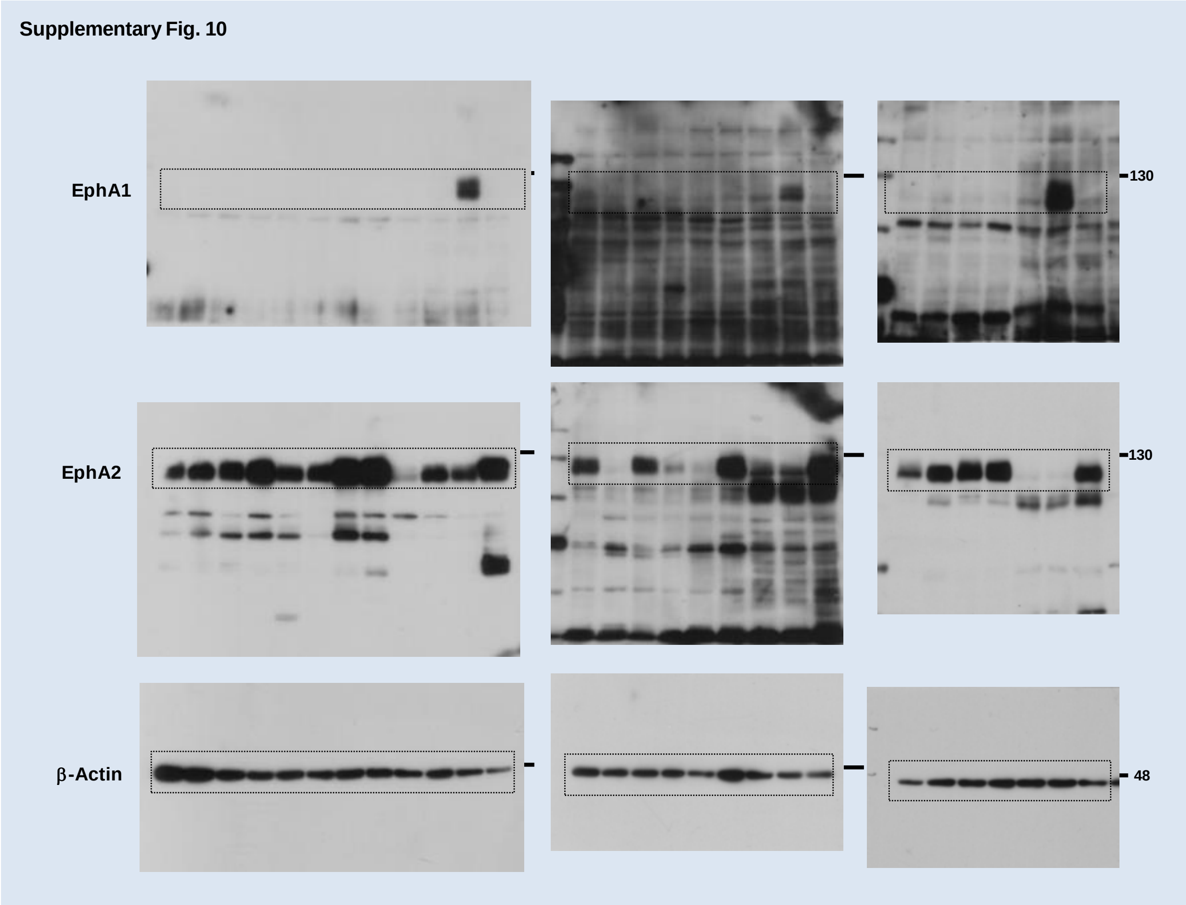

Supplementary Figure 11 Full scans of Western-blot data.

Supplementary Table

Relationship of clinicopathological and immunohistochemical detection of pRSK-negative *versus* -positive and pS-EphA2-negative/pRSK-positive *versus* pS-EphA2-positive/pRSK-positive samples. *P*-values were calculated by the two-sided Fisher's exact tests.

Variable	catagorization	pRSK			pS-EphA2/pRSK		
		negative	positive	P	−/+	+/+	P
Age (y)							
	＜60	50	28	0.893	24	4	0.781
	≧60	175	92		74	18	
Gender							
	Male	167	70	0.003	56	14	0.639
	Female	58	50		42	8	
Smoking							
	negative	66	47	0.111	37	10	0.461
	positive	136	65		55	10	
Stage							
	I	80	48	0.877	41	7	0.395
	II	34	17		12	5	
	III-IV	29	16		14	2	

Supplementary materials and methods

Antibodies and reagents

Anti-phospho-TAK1 (Thr-187) antibody was as reported previously [1]. The phospho-specific antibodies against Bad (Ser-112; #5284), MAPKAPK2 (Thr-334; #3041) and p65 (Ser-536; #3033) were purchased from Cell Signaling Technology. Antibodies against Caspase-3 (#9662) and PARP (#9542) were from Cell Signaling Technology; EphA1 (MAB3034) was from R&D Systems. For immunoblotting, all antibodies were diluted 1:1000, except phospho-p65 and EphA1, which were diluted 1:4000 and 100 ng ml−1, respectively. Recombinant human IL-1β was obtained from R&D Systems. Trametinib was purchased from AdooQ BioScience (Irvine, CA, USA).

Transfection

HeLa and A549 cells were transfected with plasmid DNAs and/or siRNAs using Lipofectamine Reagent (Life Technologies Corporation) or Lipofectamine LTX in accordance with the manufacturer's instructions, respectively. Expression vectors for human TAK1, TAB1 and TAB2 were as reported previously [2-3].

Cell viability assay

Viability of cells was determined by WST-1 Cell Counting Kit (Wako Pure Chemical Industries) in accordance with the manufacturer's instructions.

Matrigel invasion assay

The Matrigel invasion assay of MDA-MB-231 cells was performed using Transwell cell culture chambers (Corning Costar, Cambridge, Mass., USA) according to methods reported previously [4]. Polyvinylpyrrolidone-free polycarbonate filters with an 8.0-μm pore size (Nuclepore, Pleasanton, Calif., USA) were precoated with 1 μg of fibronectin on the lower surface, and then 5 μg of Matrigel was applied to this upper surface. After drying at room temperature, cells suspended in DMEM with 0.1% BSA were added to the upper compartment of the chamber and incubated at 37°C in a 5% CO_2 atmosphere. The filters were then fixed with methanol and stained with hematoxylin and eosin. After gentle rinsing with water, the remaining cells on the upper surface of the filters were removed by wiping with a cotton swab. The filters containing the stained cells that had invaded to their lower surface were counted using the mean of five windows per filter. Analysis was carried out at least three times. Quantification was performed blind to experimental conditions.

Immunohistochemistry

For immunohistochemical staining, RSK1 antibody was diluted 1:400. Nuclear and/or cytoplasmic staining was considered positive. Four semiquantitative classes were used to describe the intensity of staining; no staining, weak staining, moderate staining and strong staining. Evaluation was performed in blind manner.

Supplementary references

[1] Singhirunnusorn, P., Suzuki, S., Kawasaki, N., Saiki, I. & Sakurai, H. Critical roles of threonine 187 phosphorylation in cellular stress-induced rapid and transient activation of transforming growth factor-beta-activated kinase 1 (TAK1) in a signaling complex containing TAK1-binding protein TAB1 and TAB2. *J. Biol. Chem.* **280**,7359-7368 (2005)

[2] Sakurai, H., Miyoshi, H., Toriumi, W. & Sugita, T. Functional interactions of transforming growth factor beta-activated kinase 1 with IkappaB kinases to stimulate NF-kappaB activation. *J. Biol. Chem.* **274**, 10641-10648 (1999)

[3] Sakurai, H., Shigemori, N., Hasegawa, K. & Sugita, T. TGF-beta-activated kinase 1 stimulates NF-kappa B activation by an NF-kappa B-inducing kinase-independent mechanism. *Biochem. Biophys. Res. Commun.* **243**, 545-549 (1998)

[4] Ueno, Y. *et al.* Selective inhibition of TNF-alpha-induced activation of mitogen-activated protein kinases and metastatic activities by gefitinib. *Br. J. Cancer* **92**,1690-1695 (2005)
